# Kinesin-5 Eg5 is essential for spindle assembly and chromosome alignment of mouse spermatocytes

**DOI:** 10.1186/s13008-020-00063-4

**Published:** 2020-03-06

**Authors:** Zhen-Yu She, Ning Zhong, Kai-Wei Yu, Yu Xiao, Ya-Lan Wei, Yang Lin, Yue-Ling Li, Ming-Hui Lu

**Affiliations:** 1 Department of Cell Biology and Genetics, The School of Basic Medical Sciences, Fujian Medical University, Fuzhou, 350122 Fujian China; 2 Key Laboratory of Stem Cell Engineering and Regenerative Medicine, Fujian Province University, Fuzhou, 350122 Fujian China; 3 Fujian Obstetrics and Gynecology Hospital, Fuzhou, 350001 Fujian China; 4 Fujian Provincial Children’s Hospital, Fujian Provincial Maternity and Children’s Hospital, Affiliated Hospital of Fujian Medical University, Fuzhou, 350001 Fujian China

**Keywords:** Kinesin-5, Eg5, Spermatocyte, Meiosis, Spindle, Microtubule, Chromosome, Spermatogenesis

## Abstract

**Background:**

Microtubule organization is essential for bipolar spindle assembly and chromosome segregation, which contribute to genome stability. Kinesin-5 Eg5 is known to be a crucial regulator in centrosome separation and spindle assembly in mammalian somatic cells, however, the functions and mechanisms of Eg5 in male meiotic cell division remain largely unknown.

**Results:**

In this study, we have found that Eg5 proteins are expressed in mouse spermatogonia, spermatocytes and spermatids. After Eg5 inhibition by specific inhibitors Monastrol, STLC and Dimethylenastron, the meiotic spindles of dividing spermatocytes show spindle collapse and the defects in bipolar spindle formation. We demonstrate that Eg5 regulates spindle bipolarity and the maintenance of meiotic spindles in meiosis. Eg5 inhibition leads to monopolar spindles, spindle abnormalities and chromosome misalignment in cultured GC-2 spd cells. Furthermore, Eg5 inhibition results in the decrease of the spermatids and the abnormalities in mature sperms.

**Conclusions:**

Our results have revealed an important role of kinesin-5 Eg5 in male meiosis and the maintenance of male fertility. We demonstrate that Eg5 is crucial for bipolar spindle assembly and chromosome alignment in dividing spermatocytes. Our data provide insights into the functions of Eg5 in meiotic spindle assembly of dividing spermatocytes.

## Background

The formation of the bipolar spindle is indispensable for faithful chromosome segregation [1]. Microtubule-associated proteins, such as kinesins and dynein, are required for spindle assembly and chromosome movements during cell division [2]. Mitotic kinesins play a critical role in the regulation of microtubule organization and dynamics. In vertebrates, kinesin-5 motors are evolutionarily conserved motor proteins with plus-end-directed motility [3, 4]. In humans and *Xenopus,* kinesin-5 Eg5 forms homotetramers to bundle and slide microtubules in dividing cells [5–8].

In vertebrates, kinesin-5 Eg5 crosslinks parallel microtubules and slides antiparallel microtubules to stabilize spindle microtubules in mitosis [5, 9, 10]. Antiparallel interpolar microtubules are pushed apart by Eg5 through its plus-end-directed motility [11]. In addition, Eg5 also locates at microtubule plus-ends and promotes microtubule polymerization [12–14]. Eg5 stabilizes microtubule bundles and regulates chromosome movements [15]. Recent studies have shown that kinesin-5 acts as a molecular brake within antiparallel microtubules to regulate microtubule motions [8, 16, 17].

In human somatic cells, Eg5 is essential for spindle pole separation [3]. After Eg5 inhibition, the bipolar spindle collapses into the monopolar spindle within unseparated sister chromatids [18, 19]. Eg5 is required for the formation and maintenance of the mitotic spindle [20]. SiRNA-mediated Eg5 depletion results in the abnormalities of bipolar spindles and the formation of monoastral spindles [21]. Eg5 inhibition results in monopolar spindle and the polyploidy [22, 23].

In mammalian cells, kinesin-5 motors are essential for the establishment of spindle bipolarity [24]. The bipolar spindle is maintained by the counterbalance of kinesin-14 and kinesin-5 [25]. Kinesin-5 generates outward pushing forces to separate spindle poles [19], whereas kinesin-14 and dynein generate inward pulling forces [25]. In mammalian oocytes, Eg5 regulates meiotic spindle assembly [26–28] and poleward tubulin flux [29]. Eg5 is required for the maintenance of bipolar spindle in mouse oocytes during meiosis II [30]. However, roles and mechanisms of Eg5 in male meiotic division remain largely unknown.

To date, several chemical compounds targeting kinesin-5 Eg5 have been identified [31]. Three chemical inhibitors, including Monastrol, (*S*)-trityl-_l_-cysteine (STLC) and Dimethylenastron, are useful tools for studies about biological functions of Eg5. Eg5 inhibition activates the spindle assembly checkpoint and induces cell cycle arrest in human cancer cells [32, 33]. In prostate cancer cells, Eg5 depletion leads to growth inhibition, the G_2_/M arrest and apoptosis [22, 23].

To gain insights into the meiotic functions of kinesin-5 Eg5, we investigate the functions of Eg5 in vitro and in vivo. In this study, our data show that Eg5 proteins are expressed in mouse spermatogonia, spermatocytes and elongating spermatids. Using specific inhibitor-mediated approaches in male mouse gonads, we further reveal that Eg5 inhibition results in spindle collapse in dividing spermatocytes and the defects in spermatogenesis. In cultured spermatocytes, the inhibition of Eg5 leads to the monopolar spindles, spindle abnormalities and chromosome misalignment. Furthermore, we demonstrate that Eg5 inhibition also results in the formation of abnormal sperms and the decreased number of sperms. Eg5 is essential for the establishment of the structure of sperm head. Taken together, our data suggest that kinesin-5 Eg5 regulates spindle bipolarity and chromosome alignment in meiotic cell division, and also contributes to the maintenance of sperm numbers and male fertility.

## Results

### Eg5 proteins locate at the cytoplasm and spindle microtubules in mouse spermatogenic cells

**Fig. 1:**
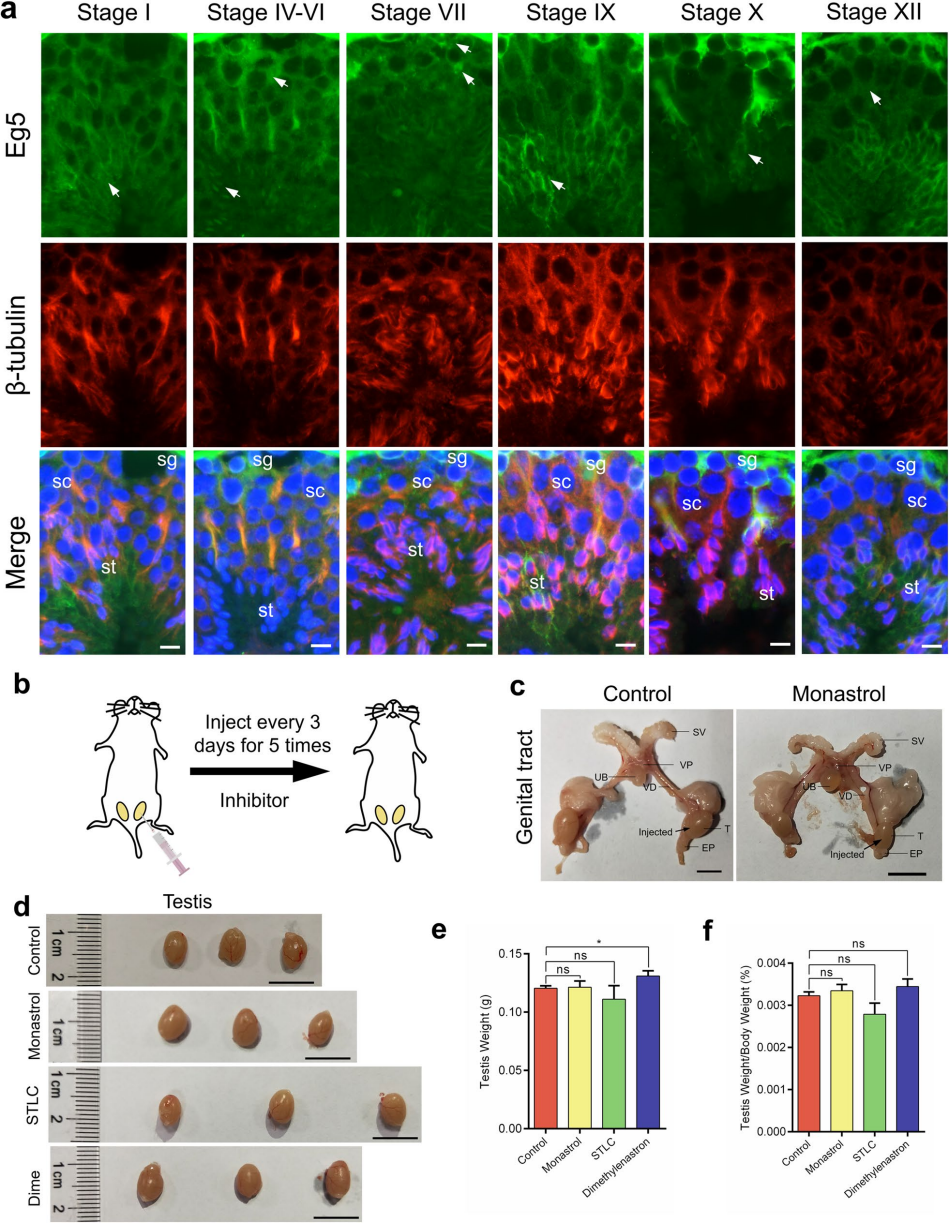
Expression pattern of Eg5 proteins in mouse testes and the establishment of the mouse model. **a** The spatiotemporal distribution of Eg5 proteins (green), β-tubulin (red) and DAPI (blue) at different stages of mouse seminiferous tubules. Representative images of stage I, IV–VI, VII, IX, X and XII were shown. Arrows indicate the fluorescent signals of Eg5 proteins. sg, spermatogonia; sc, spermatocytes; st, spermatids. Scale bar, 10 μm. **b** Graphical model of the testicular injection. Monastrol, STLC and Dimethylenastron were injected into the right testes of 6-week-old ICR mice at final concentrations of 50 μM, 10 μM and 20 μM, respectively. The inhibitors were injected every 3 days for 5 times. **c**, **d** Morphology of the male genital tract from the Control and Monastrol groups and the testes from the Control, Monastrol, STLC and Dimethylenastron groups. T (testis), SV (seminal vesicle), VP (ventral prostate), VD (vas deferens), EP (epididymis) and UB (urinary bladder). Scale bar, 10 mm. The mice from C–D were age matched. **e**, **f** Statistical analyses of the testis weight and the ratios of testis weight/body weight in the Control, Monastrol, STLC and Dimethylenastron groups. n = 8, 11, 3, 3. Student’s *t*-test. Error bars, mean ± SEM. ns, *p* > 0.05 and *, *p* < 0.05

We first used the immunofluorescence assay to investigate the distribution of Eg5 proteins in mouse testes. From stage I to XII, Eg5 proteins were expressed in the spermatogonia, the spermatocytes and the elongating spermatids (Fig. 1a, Additional file 1: Fig. S1a, b). Eg5 proteins were mainly located at the cytoplasm and partially co-localized with microtubules in the spermatogenic cells (Fig. 1a, Additional file 1: Fig. S1a). A portion of Eg5 proteins co-localized with the spindle microtubules of the dividing spermatocytes (Fig. 1a). Eg5 proteins were also enriched at the manchette of the elongating spermatids from stage IV to stage XII (Fig. 1a, Additional file 1: Fig. S1a). In addition, a portion of Eg5 proteins located at the nucleus of mature sperms at stage IV–VI (Fig. 1a). Taken together, Eg5 proteins are widely expressed in the spermatogenic cells, indicating that Eg5 may play a role in the process of mouse spermatogenesis.

Previous studies have shown that Eg5 can be inhibited by three specific inhibitors, including Monastrol [34], STLC [35] and Dimethylenastron [36]. Monastrol interacts with Eg5-ADP complex to inhibit ADP release and suppresses ATP hydrolysis through an allosteric mechanism [37, 38]. STLC tightly binds to Eg5 to disrupt the mitotic spindle during metaphase without direct influences on microtubule dynamics [34, 39, 40]. Dimethylenastron allosterically inhibits Eg5 through decreasing ADP release [41, 42]. These three cell-permeable inhibitors are effective tools for the long-term inhibition of Eg5 in vivo. We injected these inhibitors into the right testes of mice to study morphological changes in male mouse gonads after Eg5 inhibition (Fig. 1b). We compared the genital tracts and testes in the control group and the experimental groups, and found that there were no significant changes in the morphology and testis weight among the control, Monastrol, STLC and Dimethylenastron groups (Fig. 1c–f).

### Eg5 inhibition results in the defects in seminiferous tubules during mouse spermatogenesis

**Fig. 2:**
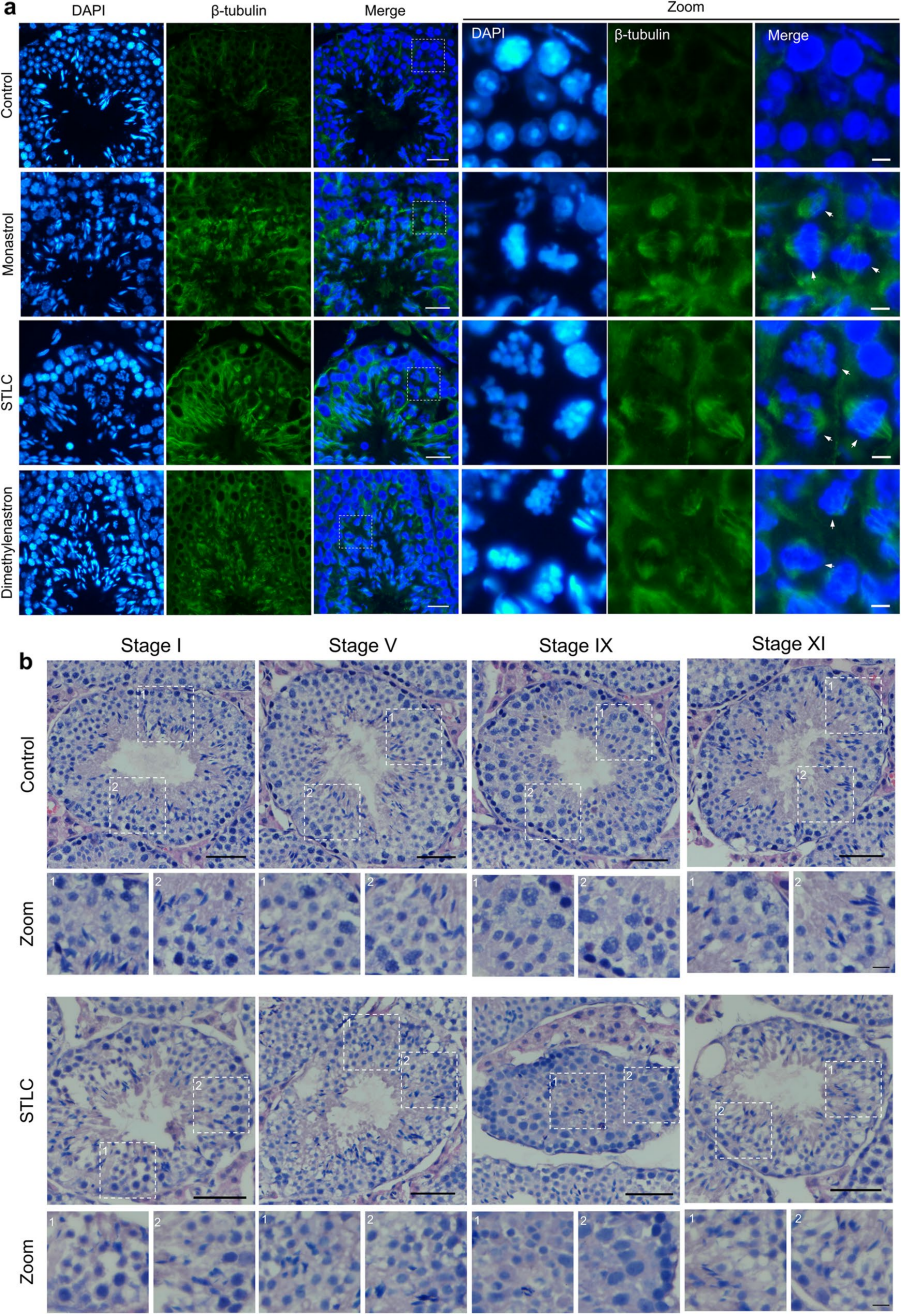
Eg5 inhibition resulted in the increase of monopolar spindles in mouse spermatocytes and the disruption of seminiferous tubules. **a** Representative images of the immunofluorescence of the seminiferous tubules in the Control, Monastrol (50 μM), STLC (10 μM) and Dimethylenastron (20 μM) groups. DAPI (blue) and β-tubulin (green). Scale bar, 25 μm. The enlarged images of metaphase-arrested spermatocytes and abnormal spermatogenic cells were shown in the zoom. The misaligned chromosomes and monopolar spindles were shown. Scale bar, 5 μm. **b** HE staining of the seminiferous tubules in the Control and STLC (10 μM) groups. Representative images of stage I, V, IX and XI were shown. Scale bar, 50 μm. The magnified images of dashed boxes were shown. Scale bar, 20 μm (Zoom)

In order to investigate the biological functions of Eg5 in mouse spermatogenesis, we then analyzed the structures of seminiferous tubules and the organizations of spermatogenic cells using immunofluorescence and HE staining. In the control group, the spermatogenic wave was organized and the spermatogenic cells were neatly arranged. However, there were several disordered seminiferous tubules and disorganized spermatogenic cells in the Monastrol, STLC and Dimethylenastron groups (Fig. 2). After Eg5 inhibition, some seminiferous tubules were slightly disordered and showed untidy margin (Additional file 1: Fig. S1c).

Strikingly, we observed the metaphase-arrested spermatocytes and the monoastral spindles in the Monastrol, STLC and Dimethylenastron groups (Fig. 2a). We found that a portion of dividing spermatocytes were arrested at metaphase. The spindle microtubules in dividing spermatocytes were severely disrupted and the chromosomes were scattered around the spindle poles after Eg5 inhibition. In dividing spermatocytes, the monoastral spindle showed a rosette-like microtubule array with two centrosomes surrounded by a ring of chromosomes (Fig. 2a).

Furthermore, Eg5 inhibition led to the disarrangement of spermatogenic cells in seminiferous tubules (Fig. 2b, Additional file 1: Fig. S2). Particularly, in several seminiferous tubules, the number of mature spermatids was greatly reduced (Fig. 2b, Additional file 1: Fig. S2). The number of metaphase-arrested cells and dead cells was significantly increased after Eg5 inhibition (Fig. 2b, Additional file 1: Fig. S2). In the Dimethylenastron-treated group, the abnormalities were similar to the STLC group but showed more severe phenotypes. Taken together, our results suggest that Eg5 is essential for the meiotic division of mouse spermatocytes through the regulation of bipolar spindle formation and chromosome alignment. In mouse testes, Eg5 inhibition leads to a significantly increase in metaphase-arrested cells and the disorganization of seminiferous tubules. In addition, Eg5 inhibition results in a decrease in the spermatids and an increase in dead cells.

### Eg5 inhibition slightly disrupts the ultrastructure of the nucleus of mouse spermatocytes

**Fig. 3:**
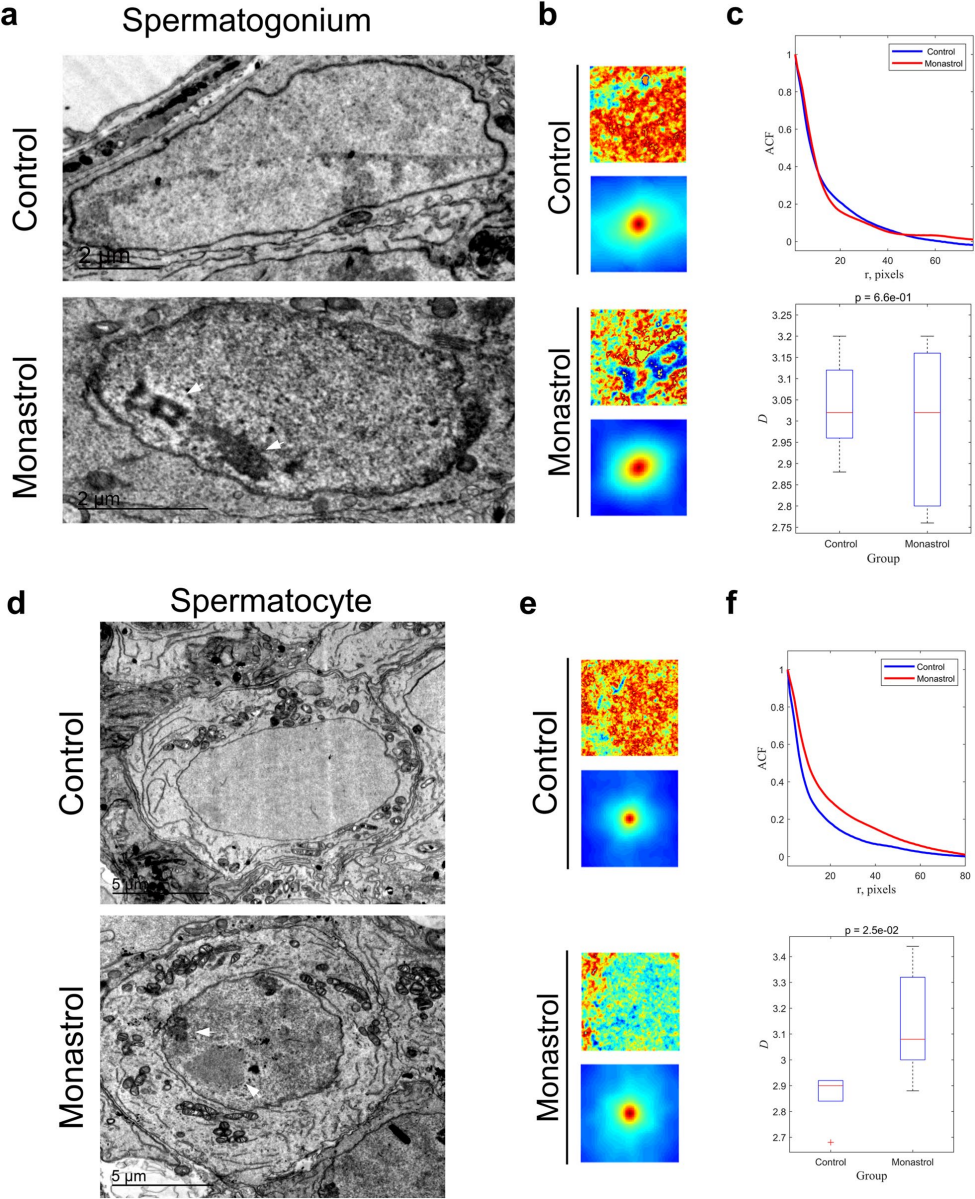
The ultrastructure of the spermatogonia and spermatocytes after the inhibition of Eg5. **a** Electron microscopic images of the spermatogonia in the Control and Monastrol groups. The 6-week-old mice were treated by Monastrol (50 μM). The arrows indicated the nucleolus and electron-dense region in the nucleus. Scale bar, 2 μm. **b** The quantifications of chromatin mass density in the spermatogonium were performed by the measurement of the value of separation *r* (pixel) in the original images (n = 6). **c** Comparisons of the average *ACF* (autocorrelation functions) and the calculated *D*-value of the spermatogonium in the Control and Monastrol groups. A boxplot indicated all *D* values corresponding to their correlation functions. **d** Electron microscopic images of the spermatocytes in the Control and Monastrol (50 μM) groups. The arrows indicated the heterochromatin in the nucleus. Scale bar, 5 μm. **e** The quantifications of chromatin mass density in the spermatocytes. The values of separation *r* (pixel) in the original images (n = 6) were shown. **f** The diagrams of the calculated *D*-value in the Control and Monastrol (50 μM) groups

To further study the detailed functions of Eg5 in mouse testes, we used electron microscopy to observe the ultrastructure of the spermatogonium and spermatocyte in the control, Monastrol, STLC and Dimethylenastron groups (Fig. 3, Additional file 1: Fig. S3). The quantification of chromatin mass density was performed in the electronic images and the heat map of two-dimensional spatial correlation function according to previous standard protocols [43]. The average *ACF* (autocorrelation functions) and a box plot of calculated *D* values were symbols of morphological distribution of chromatin mass density according to the Whittle-Matern family function [43].

In control, the distribution of chromatin in the nucleus was uniform and regular in the spermatogonium and the spermatocyte (Fig. 3a, d). However, the number of heterochromatin increased, especially in the spermatocyte after Eg5 inhibition (Fig. 3a, d, Additional file 1: Fig. S3). In the spermatocyte, the organization of the granular components, the fibrillar center and the dense fibrillary components in the nucleolus were slightly increased after Eg5 inhibition (Fig. 3d–f, Additional file 1: Fig. S3). Eg5 inhibition slightly influenced the organization of chromatins in the nucleus (Fig. 3). This indicates a potential role of Eg5 in chromatin organization and condensation during spermatogenesis. Taken together, Eg5 inhibition slightly disrupts the ultrastructure of the spermatocyte nucleus.

### Eg5 is essential for bipolar spindle formation and central spindle assembly in dividing spermatocytes

**Fig. 4:**
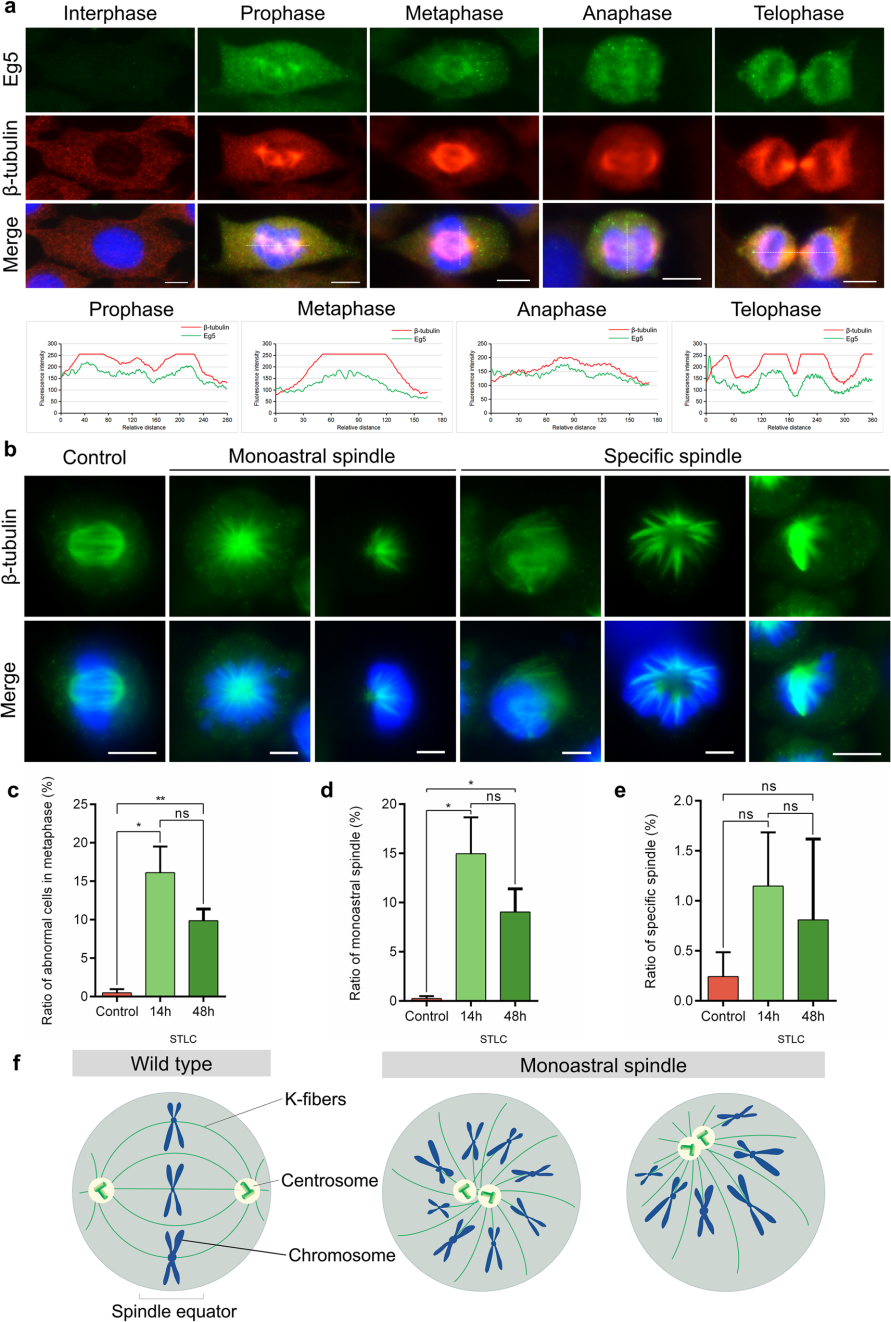
Eg5 inhibition led to monopolar spindles and cell cycle arrest at metaphase in GC-2 spd cells. **a** Representative images of the immunofluorescence of Eg5 proteins (green), β-tubulin (red) and DAPI (blue) in the cultured GC-2 spd cells. Co-localization analyses were shown by line scanning across the representative images. Scale bar, 10 μm. **b** The GC-2 spd cells were incubated with 1 μM STLC for 14 h, leading to the monoastral spindles and specific spindles at metaphase. DAPI, blue; β-tubulin, green. Scale bar, 10 μm. **c** The ratios of abnormal cells in metaphase in the Control, 14 h STLC and 48 h STLC groups (Control, 0.49 ± 0.49%; 14 h, 16.10 ± 3.41%; 48 h, 9.86 ± 1.54%). n = 3 per group. **d** The ratios of monoastral spindle in the Control, 14 h STLC and 48 h STLC groups (Control, 0.24 ± 0.24%; 14 h, 14.96 ± 3.71%; 48 h, 9.05 ± 2.34%). n = 3 per group. **e** The ratios of specific spindles in the Control, 14 h STLC and 48 h STLC groups (Control, 0.24 ± 0.24%; 14 h, 1.15 ± 0.54%; 48 h, 0.81 ± 0.81%). n = 3 per group. Student’s *t*-test. Error bars, mean ± SEM. ns, *p *> 0.05; *, *p *< 0.05 and **, *p* < 0.01. **f** Graphical model of the functions of kinesin-5 Eg5 in mouse spermatocytes. In wild-type cells, the chromosomes were orderly aligned at the spindle equator at metaphase. After Eg5 inhibition, the bipolar spindle was collapsed into the monopolar spindle and the chromosomes were misaligned around the spindle poles. Two different types of the monoastral spindles were shown

To further study how Eg5 regulates spindle assembly and chromosome alignment in spermatocytes, we then chose the GC-2 spd (ts) cell line to investigate biological functions of Eg5 in vitro. During prophase, Eg5 proteins mainly located at the spindle microtubules and showed a co-localized pattern with microtubules. At metaphase, Eg5 proteins accumulated at the kinetochore fibers. During anaphase and telophase, Eg5 proteins also located at the spindle microtubules and gradually located at the midbody (Fig. 4a).

We used immunofluorescence to examine the organization of spindle microtubules and chromosome alignment in cultured spermatocytes after Eg5 inhibition. After Eg5 inhibition, we found several types of abnormal spindles, including the monoastral spindles and the specific spindles at metaphase (Fig. 4b–f). The ratios of abnormal cells in metaphase significantly increased to 9.86–16.10% compared with 0.49% in control, demonstrating that Eg5 inhibition mainly led to metaphase arrest (Fig. 4b, c). Particularly, ratios of the monoastral spindle increased to 9.05–14.96% compared with 0.24% in control (Fig. 4d). Taken together, these results indicate that Eg5 inhibition disrupts the structures of bipolar spindles during prophase and metaphase, which contribute to the increase of the monopolar spindles and the metaphase-arrested cells.

Meanwhile, we also found that Eg5 inhibition led to the chaotic central spindle, the asymmetrical central spindle and the folded central spindle at anaphase and the multiple central spindle at telophase (Additional file 1: Fig. S4). In control, the central spindle was orderly organized into the midbody during cytokinesis. However, we found that the organization of the central spindle was influenced and the microtubules were irregular in the structures after Eg5 inhibition (Additional file 1: Fig. S4a–c). Interestingly, we found that the ratios of total telophase cells were reduced to 0.00–1.20% compared with 3.08% in control (Additional file 1: Fig. S4b), suggesting a slight decrease in the ratios of telophase cells after Eg5 inhibition. After a long-term inhibition of Eg5 in cultured spermatocytes for 48 h, the scattered chromosomes around the spindle poles at metaphase, monopolar spindles, disorganized central spindles and multiple midbodies became more apparent (Additional file 1: Fig. S4d). Taken together, these results demonstrate that Eg5 is essential for the formation of bipolar spindles at metaphase and the organization of the central spindle at telophase of dividing spermatocytes.

### Eg5 regulates chromosome alignment, segregation and chromosome stability during cell cycle

**Fig. 5:**
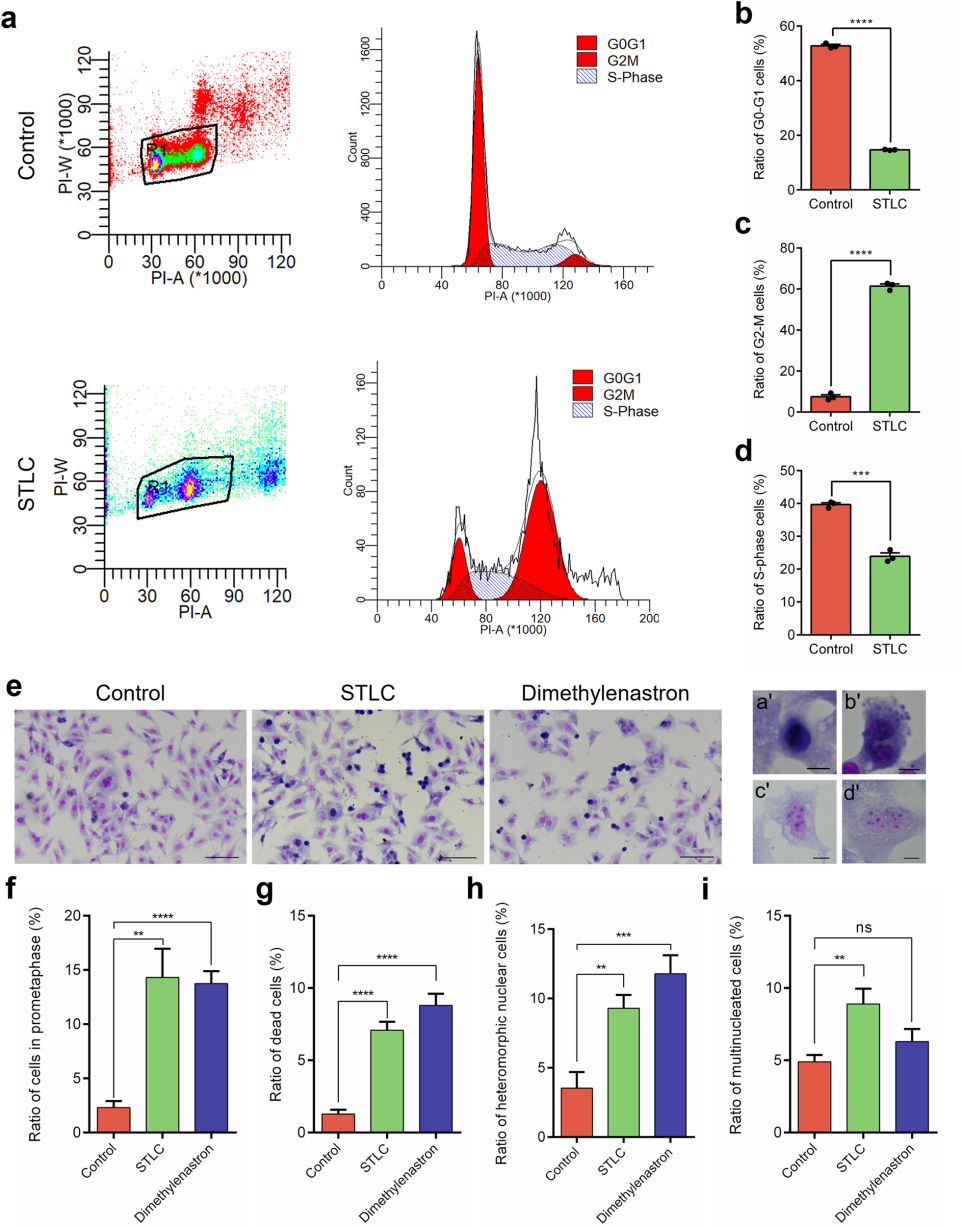
The cultured GC-2 spd cells arrested at metaphase during the cell cycle after Eg5 inhibition. **a** Cell-cycle analyses of the GC-2 spd cells incubated with 1 μM STLC for 24 h (n = 3000). The black box indicated the selected cell population of GC-2 spd cells. The G_0_/G_1_, G_2_/M and S-phase were calculated by the Modfit software and were shown in the peak figures. **b** The ratios of G_0_/G_1_ cells in the Control and STLC groups (Control, 52.78 ± 0.52%; STLC, 14.68 ± 0.10%). n = 3 per group. **c** The ratios of G_2_/M cells in the Control and STLC groups (Control, 7.52 ± 0.94%; STLC, 61.44 ± 1.04%). n = 3 per group. **d** The ratios of S-phase cells in the Control and STLC groups (Control, 39.69 ± 0.54%; STLC, 23.88 ± 1.04%). n = 3 per group. **e** Giemsa staining of the GC-2 spd cells incubated with 1 μM STLC and 1 μM Dimethylenastron for 14 h. Scale bar, 100 μm. The enlarged images indicated prometaphase cells (a’), dead cells (b’), heteromorphic nuclear cells (c’) and multinucleated cells (d’). Scale bar, 10 μm. **f** The ratios of cells in prometaphase in the Control, STLC and Dimethylenastron groups (Control, 2.31 ± 0.61%; STLC, 14.31 ± 2.64%; Dimethylenastron, 13.74 ± 1.15%). n = 7, 8, 9. **g** The ratios of dead cells in the Control, STLC and Dimethylenastron groups (Control, 1.29 ± 0.29%; STLC, 7.08 ± 0.58%; Dimethylenastron, 8.79 ± 0.81%). n = 7, 8, 9. **h** The ratios of heteromorphic nuclear cells in the Control, STLC and Dimethylenastron groups (Control, 3.53 ± 1.17%; STLC, 9.29 ± 0.97%; Dimethylenastron, 11.78 ± 1.34%). n = 7, 8, 9. **i** The ratios of multinucleated cells in the Control, STLC and Dimethylenastron groups (Control, 4.90 ± 0.47%; STLC, 8.90 ± 1.07%; Dimethylenastron, 6.29 ± 0.87%). n = 7, 8, 9. Student’s *t*-test. Error bars, mean ± SEM. ns, *p *> 0.05; **, *p *< 0.01; ***, *p *< 0.001 and ****, *p *< 0.0001

To quantify the phenotypes of metaphase-arrested cells and telophase cells after Eg5 inhibition, we used flow cytometry to analyze the ratios of spermatocytes at different stages, including the G_0_/G_1_ phase, the G_2_/M phase and the S phase (Fig. 5a). The results showed the ratios of G_0_/G_1_ phase cells markedly decreased to 14.68% in the STLC group compared with 52.78% in control (Fig. 5b). The ratios of the G_2_/M phase cells dramatically increased to 61.44% in the STLC group compared with 7.52% in control (Fig. 5c). In addition, the ratios of the S phase cells decreased to 23.88% in the STLC group compared with 39.69% in control (Fig. 5d). These results indicate that Eg5 inhibition leads to the abnormalities of cell cycle and the G_2_/M phase arrest of the GC-2 spd cells.

To further analyze the stages of GC-2 spd cells, we used the Giemsa staining to show the different stages of dividing spermatocytes (Fig. 5e–i). The results showed several abnormal phenotypes of nuclei during interphase, including the vacuolated cells, the heteromorphic nuclear cells and the multinucleated cells containing two or more nuclei (Fig. 5h, i). The ratios of heteromorphic nuclear cells significantly increased to 9.29–11.78% in the STLC and Dimethylenastron groups compared with 3.53% in control (Fig. 5h). Meanwhile, the ratios of multinucleated cells increased to 6.29–8.90% in the STLC and Dimethylenastron groups compared with 4.90% in control (Fig. 5i). These results indicate Eg5 inhibition dramatically affects the nucleus morphology at interphase and the chromosome stability during cell cycle progression.

Notably, the ratios of prometaphase cells increased to 13.74–14.31% in the STLC and Dimethylenastron groups compared with 2.31% in control (Fig. 5f), indicating that Eg5 inhibition resulted in spermatocytes arrested at prometaphase. In addition, in the STLC and Dimethylenastron groups, the ratios of dead cells significantly increased to 7.08–8.79% compared with 1.29% in control (Fig. 5g).

### Eg5 inhibition leads to decreased spermatozoa and the structural abnormalities of the sperms

**Fig. 6:**
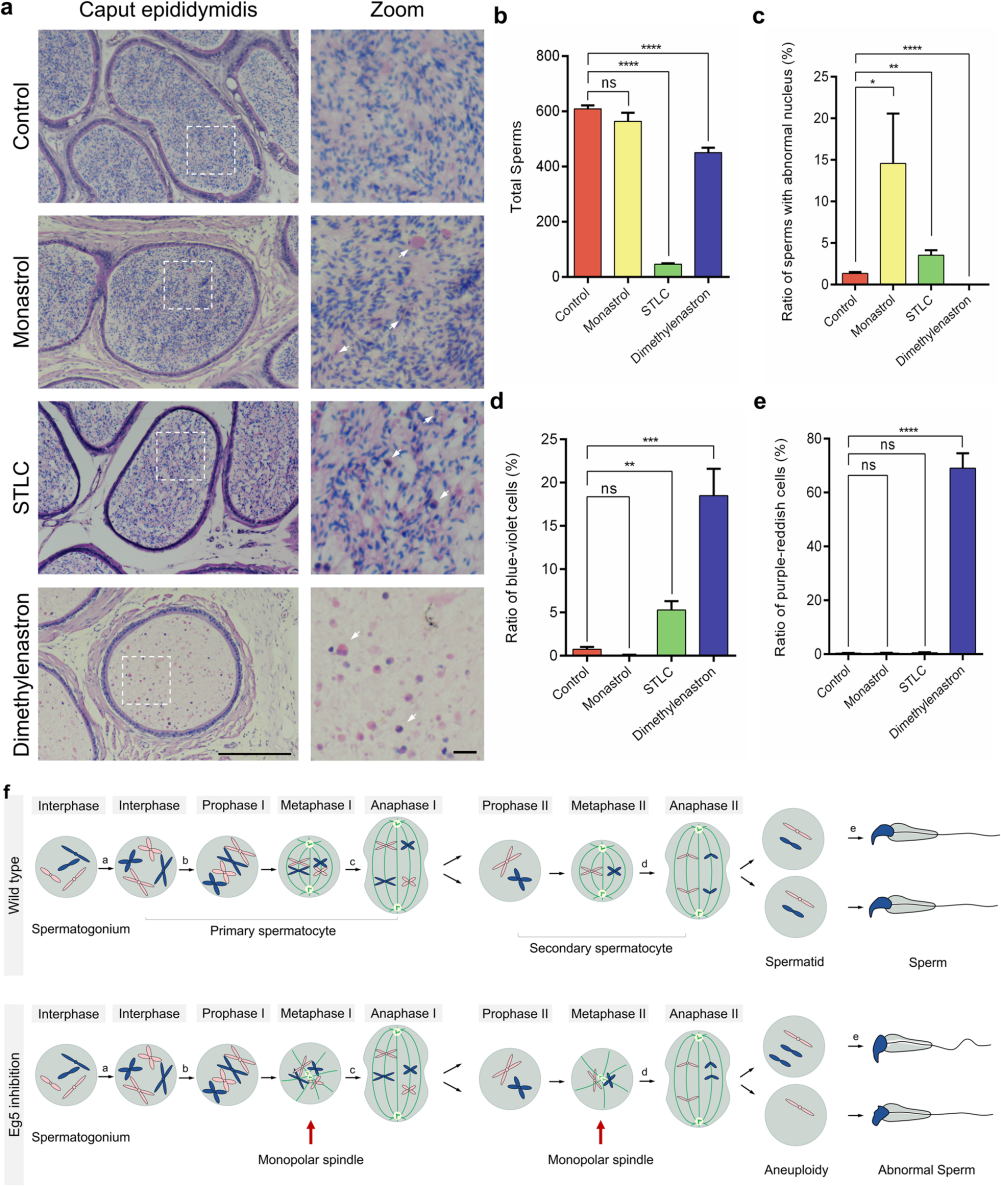
Eg5 inhibition resulted in reduced sperm counts and malformed sperm structures. **a** HE staining of the caput epididymis in the Control, Monastrol (50 μM), STLC (10 μM) and Dimethylenastron (20 μM) groups. Boxed areas were enlarged to show abnormal cells and sperms, as indicated by white arrowheads. Scale bar, 100 μm and 10 μm (Zoom). **b** Total number of the sperms from the caput epididymis per circle with a diameter of 7.9 cm in Control, Monastrol, STLC and Dimethylenastron groups (Control, 608.80 ± 12.77; Monastrol, 563.60 ± 31.10; STLC, 46.40 ± 3.33; Dimethylenastron, 450.20 ± 17.45). n = 5 per group. **c** The ratios of sperms with abnormal nucleus in the Control, Monastrol, STLC and Dimethylenastron groups (Control, 1.35 ± 0.15%; Monastrol, 14.57 ± 6.01%; STLC, 3.52 ± 0.62%; Dimethylenastron, 0.00 ± 0.00%). n = 5, 4, 5, 5. **d** The ratios of blue-violet cells in the Control, Monastrol, STLC and Dimethylenastron groups (Control, 0.73 ± 0.29%; Monastrol, 0.07 ± 0.04%; STLC, 5.28 ± 1.02%; Dimethylenastron, 18.49 ± 3.11%). n = 5 per group. **e** The ratios of purple-reddish cells in the Control, Monastrol, STLC and Dimethylenastron groups (Control, 0.36 ± 0.06%; Monastrol, 0.35 ± 0.15%; STLC, 0.51 ± 0.22%; Dimethylenastron, 69.02 ± 5.52%). n = 5 per group. Student’s *t*-test. Error bars, mean ± SEM. ns, *p *> 0.05; *, *p *< 0.05; **, *p *< 0.01; ***, *p *< 0.001; ****, *p *< 0.0001. **f** Graphical models of male meiotic division and the abnormalities of spermatogenesis after Eg5 inhibition. After Eg5 inhibition, the formation of monopolar spindles in meiosis I and meiosis II were shown. Eg5 depletion also led to the increase of aneuploid cells and abnormal sperms

To investigate the functions of Eg5 proteins in spermiogenesis, the histological analyses were used to examine the caput epididymis (Fig. 6). The total number of sperms and cells decreased by 7.42–92.38% (Fig. 6a, b). Notably, we rarely found mature sperms in the Dimethylenastron group, and sperms in other groups were disorderly arranged (Fig. 6a, c). There were blue-violet cells, purple-reddish cells and sperms with abnormal nucleus after Eg5 inhibition, which is rare in control (Fig. 6a–e). The blue-violet cells significantly increased to 5.28–18.49% in the STLC and Dimethylenastron groups compared with 0.73% in control (Fig. 6d). The purple-reddish cells, which might be dead cells, obviously increased to 69.02% in the Dimethylenastron group compared with 0.36% in control (Fig. 6e).

**Fig. 7:**
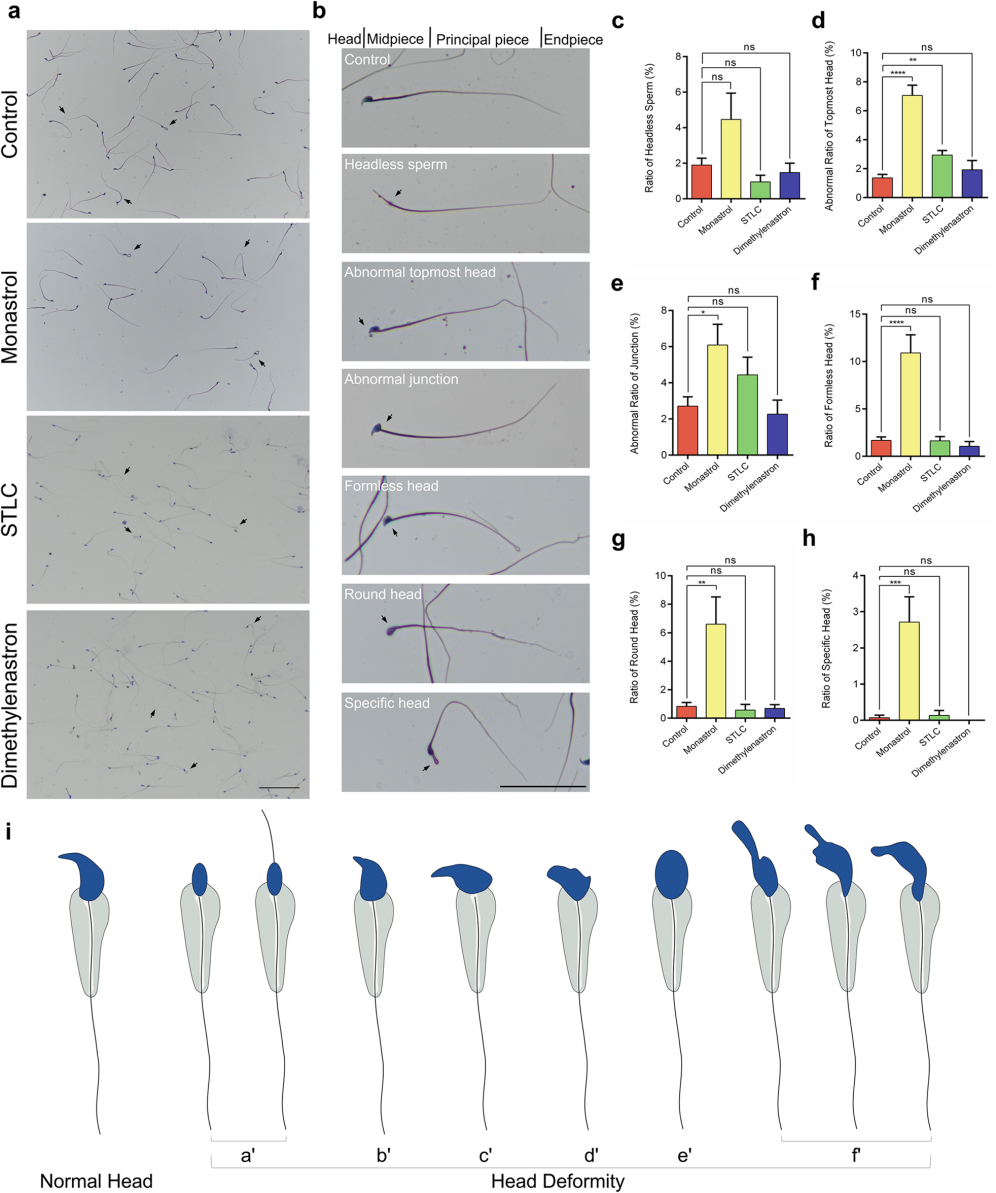
Long-term Eg5 inhibition led to different types of abnormal sperm heads. **a** HE staining of the sperms in the Control, Monastrol (50 μM), STLC (10 μM) and Dimethylenastron (20 μM). Black arrowheads pointed to the deformities of sperms. Scale bar, 100 μm. **b** Detailed morphological characteristics of abnormal sperms. Black arrowheads pointed to the deformities of sperms. Scale bar, 50 μm. **c** The ratios of headless sperm in the Control, Monastrol, STLC and Dimethylenastron groups (Control, 1.90 ± 0.38%; Monastrol, 4.46 ± 1.48%; STLC, 0.95 ± 0.38%; Dimethylenastron, 1.48 ± 0.52%). n = 11, 9, 6, 6. **d** The abnormal ratios of topmost head in the Control, Monastrol, STLC and Dimethylenastron groups (Control, 1.36 ± 0.24%; Monastrol, 7.06 ± 0.71%; STLC, 2.94 ± 0.32%; Dimethylenastron, 1.92 ± 0.64%). n = 11, 9, 6, 6. **e** The abnormal ratios of junction in the Control, Monastrol, STLC and Dimethylenastron groups (Control, 2.71 ± 0.51%; Monastrol, 6.09 ± 1.14%; STLC, 4.44 ± 0.98%; Dimethylenastron, 2.26 ± 0.79%). n = 11, 9, 6, 6. **f** The ratios of formless head in the Control, Monastrol, STLC and Dimethylenastron groups (Control, 1.68 ± 0.36%; Monastrol, 10.91 ± 1.91%; STLC, 1.64 ± 0.45%; Dimethylenastron, 1.06 ± 0.50%). n = 11, 9, 6, 6. **g** The ratios of round head in the Control, Monastrol, STLC and Dimethylenastron groups (Control, 0.83 ± 0.27%; Monastrol, 6.61 ± 1.90%; STLC, 0.57 ± 0.41%; Dimethylenastron, 0.69 ± 0.26%). n = 11, 9, 6, 6. **h** The ratios of specific head in the Control, Monastrol, STLC and Dimethylenastron groups (Control, 0.07 ± 0.07%; Monastrol, 2.72 ± 0.69%; STLC, 0.14 ± 0.14%; Dimethylenastron, 0.00 ± 0.00%). n = 11, 9, 6, 6. Student’s *t*-test. Error bars, mean ± SEM. ns, *p *> 0.05; *, *p *< 0.05; **, *p *< 0.01; ***, *p *< 0.001 and ****, *p *< 0.0001. **i** Graphical models of the abnormal sperm heads after Eg5 inhibition. The head deformity including headless sperm (a’), abnormal topmost head (b’), abnormal junction(c’), formless head (d’), round head (e’), specific head (f’) were shown

Moreover, the ratios of sperms with abnormal nuclei dramatically increased to 3.52–14.57% in the Monastrol and STLC groups compared with 1.35% in control (Fig. 6c). To further study whether the Eg5 inhibition would influence the structures of mature sperms, morphological analysis was showed on the mature sperms with short-term and long-term inhibition of Eg5 (Fig. 7, Additional file 1: Figs. S5 and S6). The short-term inhibition of Eg5 resulted in slight deformities of mature sperms (Additional file 1: Fig. S6). However, the long-term inhibition of Eg5 led to severe deformities of mature sperms (Fig. 7a, b, i, Additional file 1: Fig. S5a). The ratios of abnormal head and endpiece increased to 10.66–40.19% and 18.98–39.68% compared with 8.55% and 18.51% in control (Additional file 1: Fig. S5c, e).

There were six main deformities of sperm heads, including headless sperm (Fig. 7c), abnormal topmost head (Fig. 7d), abnormal junction (Fig. 7e), formless head (Fig. 7f), round head (Fig. 7g) and specific head (Fig. 7h). Eg5 inhibition also resulted in a slight increase in cell death in seminiferous tubules and cultured spermatocytes (Additional file 1: Fig. S7). Taken together, Eg5 inhibition causes not only the reduced counts of sperms but also the metaphase-arrested cells, the dead cells and the abnormal nuclei of sperms. Notably, the long-term inhibition contributes to more severe deformities of mature sperms.

## Discussion

To investigate the detailed roles of kinesin-5 Eg5 in male meiosis, we have inhibited Eg5 in mouse testes and have studied the effects on spindle bipolarity and chromosome alignment. We have analyzed in detail the expression pattern of Eg5 in male mouse gonads and cultured spermatocytes. We establish that Eg5 proteins are expressed in spermatogenic cells and accumulated at spindle microtubule during cell division. We show that the dividing primary spermatocytes are arrested at metaphase with monopolar spindles after Eg5 inhibition. The disruption of spermatogenic waves in mouse gonads may be caused by metaphase-arrested cells (Fig. 6f).

Eg5 is a homotetrameric plus end-directed kinesin that can crosslink and slide antiparallel microtubules [44, 45]. Eg5 motors push microtubules apart and regulate the arrangement of spindle microtubules, bipolar spindle formation and poleward microtubule flux [4, 19, 46]. Eg5 depletion or inhibition suppresses bipolar spindle formation and arrests cells at metaphase with unseparated centrosomes [5, 47]. A recent study has also indicated that kinesin-5 Eg5 proteins were expressed in spermatogonia and spermatocytes, which is consistent with our results. However, in this study, Eg5 proteins were expressed at very low level in haploid spermatocytes and Sertoli cells, which requires further validations [48]. In mammalian cells, Eg5 inhibition leads to severe defects in spindle assembly during mitosis [18, 49]. Our results highlight the important roles of kinesin-5 Eg5 in centrosome separation and bipolar spindle assembly in male meiotic division. We find that Eg5 inhibition in mouse testes results in monopolar spindle formation in primary spermatocytes and finally leads to the metaphase arrest of dividing spermatocytes.

Eg5 inhibition results in the hypoacetylation of α-tubulin, the disruption of microtubule stability, indicating that Eg5 plays a role in female meiotic maturation through mediating cytoskeleton dynamics [50]. The type of spindle formation in the oocyte is acentrosomal spindle formation. The oocytes do not contains centrosomes in many animals, including humans, mice, *Xenopus*, *Drosophila*, and *C. elegans* [51]. But the spermatocytes contain centrosomes, which suggests that the spindle formation of spermatocytes and oocytes is different [52]. However, the mechanisms of spindle formation in spermatocytes are unclear. Our data first time demonstrate that Eg5 is crucial for spindle microtubule assembly and the maintenance of spindles in dividing spermatocytes. In the absence of Eg5, the spindle microtubules in spermatocytes become highly disorganized. These results suggest that Eg5 is required for both centrosome separation and microtubule organization in spermatocytes (Fig. 6f).

Eg5 inhibition leads to the collapse of meiosis II spindles and chromosome missegregation in mammalian oocytes [30]. Eg5 is expressed in all developmental stages during porcine oocyte meiosis, and its inhibition strongly delays meiotic progression due to the reduction of Cdc2 phosphorylation with its dose-dependent property [53]. Previous studies have revealed that kinesin-5 motors regulate chromosome alignment in mitotic cells. The spindle assembly, chromosome-spindle attachment and chromosome segregation are slightly different between mitosis and meiosis [52]. The key difference between mitosis and meiosis I is that homologous chromosome are segregated in meiosis I and sister chromatids are segregated in mitosis [54]. Our results demonstrate that Eg5 inhibition results in chromosome misalignment in primary spermatocytes during meiosis I. The chromosomes are positioned around the spindle poles due to the failure of centrosome separation in meiosis. We also find that the chromosome misalignment caused by Eg5 inhibition finally leads to the formation of multinucleated cells and aneuploid cells. Taken together, kinesin-5 Eg5 is essential for chromosome alignment and the maintenance of chromosome stability in meiosis.

Recent studies have suggested that the outward pushing forces generated by kinesin-5 Eg5 can be antagonized by minus-end-directed motors, including kinesin-14 and dynein [55, 56]. Kinesin-14 KIFC1, kinesin-5 Eg5, KIF15 and dynein work together to form a force balance in the spindle [57, 58]. In mammalian cells, bipolar spindle assembly is controlled by many motor proteins and microtubule-associated proteins. For example, dynein can generate a pulling force through the astral microtubules [59] and kinetochore proteins can push centrosomes apart [60]. In this study, we show that kinesin-5 Eg5 is important for the bipolar spindle assembly in the meiotic division of spermatocytes. However, due to the complex environments in male meiosis, the detailed mechanisms of spindle assembly in meiosis remain largely unknown. In future, it is worthy to study the detailed mechanisms of kinesin motors in the force balance of meiotic spindles.

We find that Eg5 inhibition results in the decreased number of elongating spermatids and mature sperms in seminiferous tubules and the epididymis. The long-term inhibition of Eg5 in mouse testes can lead to the formation of monopolar spindle and metaphase-arrested cells, which contributes to the decreased number of the spermatids and sperms. These results suggest that Eg5 is essential for the normal development of mature sperms and the maintenance of male fertility. Previous studies have found that STLC and Monastrol bind to the same pocket on Eg5 motor domain to inhibit Eg5 motor activity and finally result in apoptosis in cancer cells. Several Eg5 inhibitors induce apoptosis in different cells and tumors [37, 61]. In HeLa cells, STLC triggers prolonged mitotic arrest and subsequent apoptosis through the activation of the intrinsic apoptotic pathway [62]. Our results also show that a portion of dead cells in seminiferous tubules after Eg5 inhibition. The prolonged cell cycle arrest of spermatocytes and cell death may be main reasons of the decrease of the mature sperms.

Interestingly, we also find several types of abnormalities in the sperm nuclei, including headless sperm, abnormal topmost head, abnormal junction, formless head, round head and specific head. These results suggest Eg5 plays a role in the maintenance of the sperm nucleus. The abnormal structure of sperm nucleus may be caused by misaligned chromosomes in meiotic division and the aneuploidy cells.

## Conclusions

In summary, we find that kinesin-5 Eg5 proteins are widely expressed in mouse spermatogonia, spermatocytes and spermatids. We further demonstrate that Eg5 inhibition leads to spindle collapse and metaphase arrest in dividing spermatocytes. In cultured GC-2 spd cells, the inhibition of Eg5 leads to the monopolar spindles, spindle abnormalities and chromosome misalignment in cell division. These results demonstrate that Eg5 is crucial for bipolar spindle assembly and chromosome alignment in dividing spermatocytes. Furthermore, Eg5 inhibition leads to the abnormalities in sperm nucleus and the reduced sperm numbers. We demonstrate that Eg5 is essential for the maintenance of sperm heads. In conclusion, our data suggest that Eg5 is crucial for spindle bipolarity and chromosome alignment in meiotic cell division. Kinesin-5 Eg5 mediates the maintenance of sperm number and male fertility.

## Methods

### Animals and ethics

The male ICR mice were purchased from the Wu-Si Experimental Animals Center (Fuzhou, China). All animal experiments were approved by the Institutional Animal Care and Use Committee at Fujian Medical University, China (protocol No. SYXK 2016-0007). All animal experiments were performed according to the federal and institutional guidelines for the Care and Use of Laboratory Animals.

### Antibodies and reagents

The primary antibodies used in this study are listed as follows: rabbit β-tubulin monoclonal antibody (Beyotime, Cat. AF1216, 1:250), mouse Eg5 monoclonal antibody (A-2) (Santa Cruz Biotechnology, Cat. sc-365593, 1:100), mouse tubulin monoclonal antibody (Beyotime, Cat. AT819, 1:500). The secondary antibodies used in this study are listed as follows: Alexa Fluor 488-labeled goat anti-rabbit IgG (H + L) (Beyotime, Cat. A0423, 1:500), Alexa Fluor 488-labeled goat anti-mouse IgG(H + L) (Beyotime, Cat. A0428, 1:500), Alexa Fluor 555-labeled donkey anti-mouse IgG (H + L) (Beyotime, Cat. A0460, 1:500) and Alexa Fluor 555-labeled donkey anti-rabbit IgG(H + L) (Beyotime, Cat. A0453, 1:500). The nuclei were stained by the DAPI solution (4, 6-diamino-2-phenyl indole) (Beyotime, Cat. C1006).

### Cell culture and tissue treatment

GC-2 spd (ts) cells (ATCC No. CRL-2196) were purchased from American Type Culture Collection. Cells were maintained and cultured in Dulbecco Modified Eagle’s Medium (DMEM/high glucose, Hyclone, Cat. SH30022.01) supplemented with 10% fetal bovine serum (FBS, Every green, Cat. 11011-8611) and 1% penicillin/streptomycin (MP Biomedicals, Cat. 1670249). Cells were cultured in a humidified incubator with 5% CO_2_ at 37 °C (Thermo Fisher Scientific).

For Eg5 inhibition, the specific inhibitors Monastrol (Santa Cruz Biotechnology, Cat. sc-202710), STLC (Santa Cruz Biotechnology, Cat. sc-202799) and Dimethylenastron (Santa Cruz Biotechnology, Cat. sc-221576) were used. For the long-term Eg5 inhibition in mouse testes, these specific inhibitors were injected 5 times in total with 3 days each injection, respectively. For Eg5 inhibition in cultured cells, the inhibitors were added into the culture medium. Then cells were cultured for 6–48 h as indicated in figure legends, respectively. The final concentrations of each specific inhibitor are listed as follows.

The concentrations of Monastrol [19, 40], STLC [37], and Dimethylenastron [41, 63] were chosen according to previous studies. For Eg5 inhibition in mouse testes, Monastrol (50 μM), STLC (10 μM) and Dimethylenastron (20 μM) were used, respectively. For Eg5 inhibition in the GC-2 spd cells, Monastrol (50 μM), STLC (1 μM) and Dimethylenastron (1 μM) were used, respectively. For the short-term Eg5 inhibition in mature sperms, the semen of untreated 6-month-old male mice was incubated with Monastrol (50 μM) at 30 °C for 4 h and 24 h, respectively.

### Immunofluorescence and confocal microscopy

For immunofluorescence, 6 μm-thick cryosections of mouse testes were fixed with 4% PFA/PBS at room temperature for 10 min. After permeabilizing with 0.25% Triton X-100/PBS at room temperature for 10 min, the sections were washed by PBS for three 5 min. The sections were blocked in the blocking solution (0.05% Tween-20 and 0.5 g/ml BSA in PBS) at room temperature for 1 h. The sections were incubated with the primary antibodies (diluted by 1% BSA/PBS) at 4 °C overnight. After washing with PBS for three 5 min, the sections were incubated with the secondary antibodies (diluted by 1% BSA/PBS) at room temperature for 2 h in the dark. The sections were washed by PBS for three 5 min and stained by DAPI staining solution (Beyotime, Cat. C1006). The anti-fade mounting medium (Beyotime, Cat. P0126) was used for mounting. The images were captured using a Nikon Ti-S microscope (Nikon Ti-S2) equipped with a Plan Apochromat 20×/0.40 objective (Nikon) and a NA 40×/0.75 objective (Nikon).

### HE and Giemsa staining

For HE staining, the cryosections were stained by Mayer’s hematoxylin for 5 min and washed by tap water for 5 min. The samples were incubated in ethanol hydrochloride for 3 s, and then incubated in lithium carbonate for 5 s. The samples were stained by 1% eosin for 5 min. The samples were dehydrated in 95% ethanol for 2 min, and then incubated in xylene for 10 min. The samples were sealed with neutral balsum.

For Giemsa staining, the samples were fixed in methanol or 4% paraformaldehyde for 10 min and then rinsed in distilled water for 5 min. The samples were stained by 10% (w/v) Giemsa staining solution (pH6.8) at room temperature for 15 min and then rinsed in distilled water for 10 min. The samples were sealed with distilled water.

The images were captured by Nikon Ti-S optical microscope equipped with a Plan Apochromat 20×/0.40 objective (Nikon) and a NA 40×/0.75 objective (Nikon). The images were analyzed using Image J software.

### Flow cytometry

The cultured cells were treated with 0.25% trypsin–EDTA at 37 °C for 10 min to dissociate cells. Cells were harvested for cell cycle analysis. The samples were fixed in 70% ethanol at 4 °C for 16 h and incubated with the staining solution (0.1% Triton X-100/PBS, RNase A and propidium iodide; Beyotime Cat. C1052) at 37 °C for 2 h. The flow cytometric analyses were performed by a flow cytometer (BD Bioscience, FACS Canto TM II). The flow cytometric results were analyzed and visualized using the Modfit LT32 software (Verity Software House).

### TUNEL assay

For detection of cell apoptosis, the 6 μm cryosections of mouse testes or cultured cells were fixed with 4% paraformaldehyde for 1 h and then washed by PBS for 10 min. After incubating with 0.5% Triton X-100 in PBS at room temperature for 5 min, the samples were stained by one step TUNEL apoptosis assay kit (Beyotime, Cat. C1086) according to the manufacturer’s protocol. The nuclei were stained by DAPI (Beyotime, Cat. C1006).

### Electron microscopy and analysis

Mouse testes were dissected into 1 mm^3^ pieces and incubated in 0.1 M PBS with 3% glutaraldehyde and 1.5% polyformaldehyde for 48 h. The testes were then incubated in 1% hydrazine solution and then washed by 0.1 M PBS. After dehydrating in gradient alcohol, 90–100% acetone and 100% acetone epoxy resin at room temperature for 2 h, the samples were embedded in epoxy resin at 35 °C for 3 h, then at 45 °C for 12 h, and at 60 °C for 2 days. The 90–100 nm ultrathin sections were obtained using an ultramicrotome (Leica EM UC-7). After staining in uranium acetate for 20 min, the samples were stained with lead citrate for 5 min. The electron images were captured using an electron microscope (FEI, Tecnai G2). The analyses of chromatin density in the nucleus were performed using a script (https://github.com/barouxlab/ChromDensityNano) from the GitHub website by the MATLAB2019a software according to the standard protocol [43].

### Statistical analysis

Data were shown as the mean ± SEM, unless indicating otherwise. All experiments were performed at least three times. For the analysis of immunofluorescence intensity, the fluorescent images were analyzed by line scan and plot analysis using Image J software (NIH). Data were analyzed by the two-tailed unpaired Student’s *t* test using the GraphPad Prism 6.0 software (GraphPad). The stages were defined according to the groups of germ cells at particular phases and the nuclear shapes in seminiferous tubules following the standard guidelines [64–66]. Each stage of cross-sectioned tubule was classified according to the head shape of the spermatids and the degree of nuclear condensation. Statistically significant results were indicated as follows: ns, not significant; *, *p* < 0.05; **, *p* < 0.01; ***, *p* < 0.001; ****, *p* < 0.0001. It was considered significant that *p* values was less than 0.05.

## Supplementary information

**Additional file 1: Fig. S1.** Immunofluorescence of Eg5 proteins at Stage IV–VI and XI and HE staining of mouse seminiferous tubules after Eg5 inhibition. Related to Figs. 1 and 2. **a** Spatiotemporal positioning of Eg5 proteins (green), β-tubulin (red) and DAPI (blue) during mouse spermatogenesis. sc, spermatocyte; st, spermatid. Scale bar, 10 μm. **b** Negative control of Alexa-488 (green), Alexa-555 (red) and DAPI (blue) in seminiferous tubule. The primary antibodies were not added in the negative control. Scale bar, 10 μm. (C) HE staining of seminiferous tubules in the Control, Monastrol, STLC and Dimethylenastron groups. The boxed areas were enlarged to show blurred boundaries of seminiferous tubules. Scale bars, 100 μm and 25 μm (Zoom). **Fig. S2.** Eg5 inhibitions resulted in the disorganization of seminiferous tubules and altered cell populations. Related to Fig. 2. HE staining of seminiferous tubules in the Monastrol (50 μM) and Dimethylenastron (20 μM) groups. Boxed areas were enlarged to show abnormalities of spermatogenic cells. Representative images of stage I, V, IX and XI were shown. Scale bars, 50 μm and 20 μm (Zoom). **Fig. S3.** The ultrastructure of the spermatogonium and spermatocytes in the STLC and Dimethylenastron group. Related to Fig. 3. **a** Electron microscopic images of the spermatogonium in the STLC and Dimethylenastron groups. Scale bar, 2 μm. **b** The quantifications of chromatin mass density in the spermatogonium (n = 6). **c** Comparisons of the average *ACF* and *D*-value of the spermatogonium in the STLC and Dimethylenastron groups. A boxplot indicated all *D* values corresponding to their correlation functions. **d** Electron microscopic images of the spermatocytes in the STLC and Dimethylenastron group. Scale bar, 2 μm. **e** The quantifications of chromatin mass density in the spermatocytes in the STLC and Dimethylenastron groups. **f** The diagrams of *D*-values in the STLC and Dimethylenastron groups. **Fig. S4.** Eg5 inhibition results in microtubule disorganization in spindle microtubules in the GC-2 spd cells. Related to Fig. 4. **a** The GC-2 spd cells were cultured with 1 μM STLC for 14 h, leading to chaotic (a’), asymmetrical (b’) and folded (c’) central spindles during telophase. DAPI (blue), β- tubulin (green). Scale bar, 10 μm. **b** The ratios of total telophase cells in the Control, 14 h STLC and 48 h STLC groups (Control, 3.08 ± 1.04%; 14 h, 1.20 ± 0.90%; 48 h, 0.00 ± 0.00%). n = 3 per group. **c** The ratios of abnormal cells in telophase in the Control, 14 h STLC and 48 h STLC groups (Control, 0.00 ± 0.00%; 14 h, 0.99 ± 0.99%; 48 h, 0.00 ± 0.00%). n = 3 per group. Student’s *t*-test. Error bars, means ± SEM. ns, *p* > 0.05; *, *p* < 0.05. **d** The GC-2 spd cells were cultured with 1 μM STLC for 48 h, leading to monoastral spindle in metaphase (d’), asymmetrical central spindle in anaphase (e’) and multipolar central spindle in telophase (f’). DAPI (blue), β-tubulin (green). Scale bar, 10 μm. **Fig. S5.** Long-term Eg5 inhibition resulted in various types of abnormal sperms. Related to Fig. 7. **a** Detailed morphological characteristics of abnormal sperms. Black arrowheads pointed to the deformities of sperms. Scale bar, 50 μm. **b** The ratios of abnormal sperm head in the Control, Monastrol, STLC and Dimethylenastron groups. (Control, group = 11, n = 101; Monastrol, group = 9, n = 320; STLC, group = 6, n = 80; Dimethylenastron, group = 6, n = 318). **c** The abnormal ratios of head in the Control, Monastrol, STLC and Dimethylenastron groups (Control, 8.55 ± 0.98%; Monastrol, 37.86 ± 5.80%; STLC, 10.66 ± 1.77%; Dimethylenastron, 40.19 ± 4.15%). n = 11, 9, 6, 6. **d** The abnormal ratios of midpiece in the Control, Monastrol, STLC and Dimethylenastron groups (Control, 20.93 ± 2.25%; Monastrol, 25.38 ± 2.61%; STLC, 20.94 ± 1.39%; Dimethylenastron, 22.05 ± 1.21%). n = 11, 9, 6, 6. **e** The abnormal ratios of endpiece in Control, Monastrol, STLC and Dimethylenastron groups (Control, 18.51 ± 0.99%; Monastrol, 39.68 ± 2.75%; STLC, 23.09 ± 2.63%; Dimethylenastron, 18.98 ± 3.05%). n = 11, 9, 6, 6. **f** The ratios of curving endpiece in the Control, Monastrol, STLC and Dimethylenastron groups (Control, 9.57 ± 0.90%; Monastrol, 29.64 ± 2.14%; STLC, 17.75 ± 1.97%; Dimethylenastron, 11.43 ± 2.49%). n = 11, 9, 6, 6. Student’s *t*-test. Error bars, means ± SEM. ns, *p* > 0.05; ***, *p* < 0.001; ****, *p* < 0.0001. **Fig. S6.** Short-term Eg5 inhibition lead to mild phenotypes in mature sperms. Related to Fig. 7**a**, **d** HE staining of mature sperms in the Control and Monastrol groups. The semen of untreated 6-month-old mouse was incubated by 50 μM Monastrol at 30 ℃ for 4 h and 24 h, respectively. Black arrowheads pointed to the deformities of sperms. Scale bar, 100 μm. **b, e** Detailed morphological characteristics of abnormal sperms at 30 ℃ for 4 h and 24 h. Scale bar, 25 μm. **c** The abnormal ratios of the midpiece (Control, 15.64 ± 2.87%; Monastrol, 15.87 ± 3.05%) and the endpiece (Control, 15.87 ± 3.05%; Monastrol, 35.65 ± 2.09%) in the Control and Monastrol groups. 30 ℃ for 4 h. n = 3 per group. **f** The abnormal ratios of the midpiece (Control, 19.15 ± 1.83%; Monastrol, 21.09 ± 3.44%) and the endpiece (Control, 35.10 ± 2.99%; Monastrol, 40.97 ± 3.86%) in the Control and Monastrol group. 30 ℃ for 24 h. n = 3 per group. Student’s *t*-test. Error bars, means ± SEM. ns, *p* > 0.05 and **, *p* < 0.01. **Fig. S7.** Cell apoptosis analyses of seminiferous tubules and GC-2 spd cells after Eg5 inhibition. Related to Figs. 2, 4, 5 and 6. **a** TUNEL analyses of seminiferous tubules treated by Monastrol (50 μM, 2 weeks). **b** Ratio of TUNEL positive cell per tubule. Control, 3.17 ± 0.48; Monastrol, 6.17 ± 0.60. n = 6. Student’s *t*-test. Error bars, means ± SEM. ***, *p* < 0.001. **c** TUNEL analyses of GC-2 spd cells cultured by STLC (1 μM, 14 h) and Dimethylenastron (1 μM, 14 h). DAPI (blue), TUNEL (green). Scale bar, 50 μm. **d** Ratio of TUNEL positive cells in the control, STLC and Dimethylenastron groups. Control, 1.00 ± 0.25%; STLC, 2.83 ± 0.54%; Dimethylenastron, 4.50 ± 0.76%. n = 200, group = 6. Student’s *t*-test. Error bars, means ± SEM. *, *p* < 0.01; ***, *p* < 0.001.

## Data Availability

All data generated or analyzed during this study are included in this published article.

## Abbreviations

ADP: Adenosine diphosphate
BSA: Albumin from bovine serum
DAPI: 4, 6-diamino-2-phenyl indole
PBS: Phosphate buffer saline
STLC: (*S*)-trityl-_l_-cysteine
