# Supplementary material for: Kinesin-5 Eg5 is essential for spindle assembly and chromosome alignment of mouse spermatocytes
Source: Cell Div. 2020 Mar 6;15:6. doi: 10.1186/s13008-020-00063-4 (PMC7060529; doi:10.1186/s13008-020-00063-4)
Supplement: Supplementary file 1 — Additional file 1: Fig. S1. Immunofluorescence of Eg5 proteins at Stage IV–VI and XI and HE staining of mouse seminiferous tubules after Eg5 inhibition. Related to Figs. 1 and 2. a Spatiotemporal positioning of Eg5 proteins (green), β-tubulin (red) and DAPI (blue) during mouse spermatogenesis. sc, spermatocyte; st, spermatid. Scale bar, 10 μm. b Negative control of Alexa-488 (green), Alexa-555 (red) and DAPI (blue) in seminiferous tubule. The primary antibodies were not added in the negative control. Scale bar, 10 μm. (C) HE staining of seminiferous tubules in the Control, Monastrol, STLC and Dimethylenastron groups. The boxed areas were enlarged to show blurred boundaries of seminiferous tubules. Scale bars, 100 μm and 25 μm (Zoom). Fig. S2. Eg5 inhibitions resulted in the disorganization of seminiferous tubules and altered cell populations. Related to Fig. 2. HE staining of seminiferous tubules in the Monastrol (50 μM) and Dimethylenastron (20 μM) groups. Boxed areas were enlarged to show abnormalities of spermatogenic cells. Representative images of stage I, V, IX and XI were shown. Scale bars, 50 μm and 20 μm (Zoom). Fig. S3. The ultrastructure of the spermatogonium and spermatocytes in the STLC and Dimethylenastron group. Related to Fig. 3. a Electron microscopic images of the spermatogonium in the STLC and Dimethylenastron groups. Scale bar, 2 μm. b The quantifications of chromatin mass density in the spermatogonium (n = 6). c Comparisons of the average ACF and D-value of the spermatogonium in the STLC and Dimethylenastron groups. A boxplot indicated all D values corresponding to their correlation functions. d Electron microscopic images of the spermatocytes in the STLC and Dimethylenastron group. Scale bar, 2 μm. e The quantifications of chromatin mass density in the spermatocytes in the STLC and Dimethylenastron groups. f The diagrams of D-values in the STLC and Dimethylenastron groups. Fig. S4. Eg5 inhibition results in microtubule disorganization in spindl [file 13008_2020_63_MOESM1_ESM.pdf]

Supplemental information

Kinesin-5 Eg5 is essential for spindle assembly and chromosome alignment of mouse spermatocytes

Zhen-Yu She[1,2,#,*], Ning Zhong[1,#], Kai-Wei Yu[1], Yu Xiao[1], Ya-Lan Wei[3,4], Yang Lin[1], Yue-Ling Li[1], Ming-Hui Lu[1]

Additional File 1

Additional file 1: Fig. S1

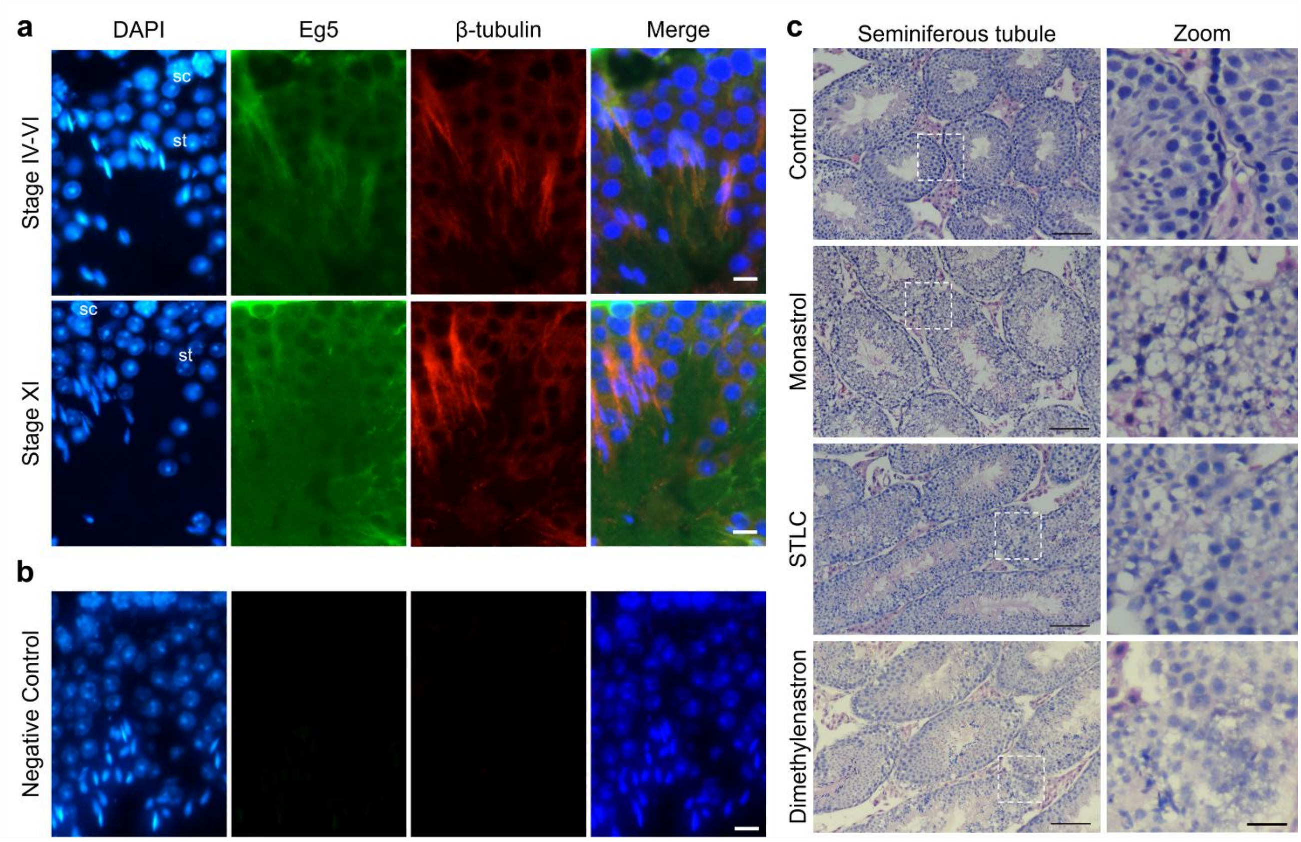

Additional file 1: Fig. S1. Immunofluorescence of Eg5 proteins at Stage IV-VI and XI and HE staining of mouse seminiferous tubules after Eg5 inhibition. Related to Fig. 1 and 2. **a** Spatiotemporal positioning of Eg5 proteins (green), β-tubulin (red) and DAPI (blue) during mouse spermatogenesis. sc, spermatocyte; st, spermatid. Scale bar, 10 μm. **b** Negative control of Alexa-488 (green), Alexa-555 (red) and DAPI (blue) in seminiferous tubule. The primary antibodies were not added in the negative control. Scale bar, 10 μm. (C) HE staining of seminiferous tubules in in the Control, Monastrol, STLC and Dimethylenastron groups. The boxed areas were enlarged to show blurred boundaries of seminiferous tubules. Scale bars, 100 μm and 25 μm (Zoom).

Additional file 1: Fig. S2

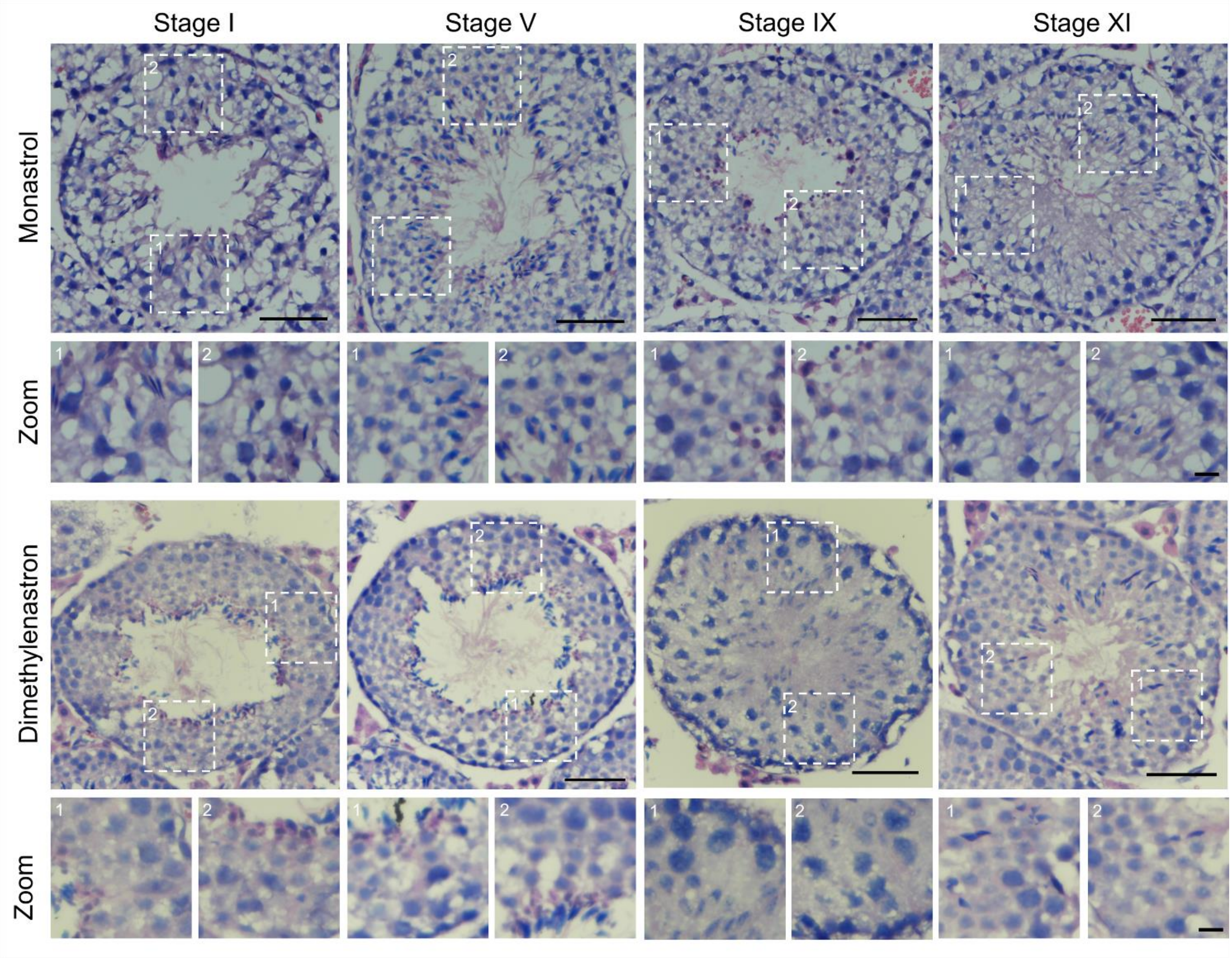

Additional file 1: Fig. S2. Eg5 inhibitions resulted in the disorganization of seminiferous tubules and altered cell populations. Related to Fig. 2. HE staining of seminiferous tubules in the Monastrol (50 μM) and Dimethylenastron (20 μM) groups. Boxed areas were enlarged to show abnormalities of spermatogenic cells. Representative images of stage I, V, IX and XI were shown. Scale bars, 50 μm and 20 μm (Zoom).

Additional file 1: Fig. S3

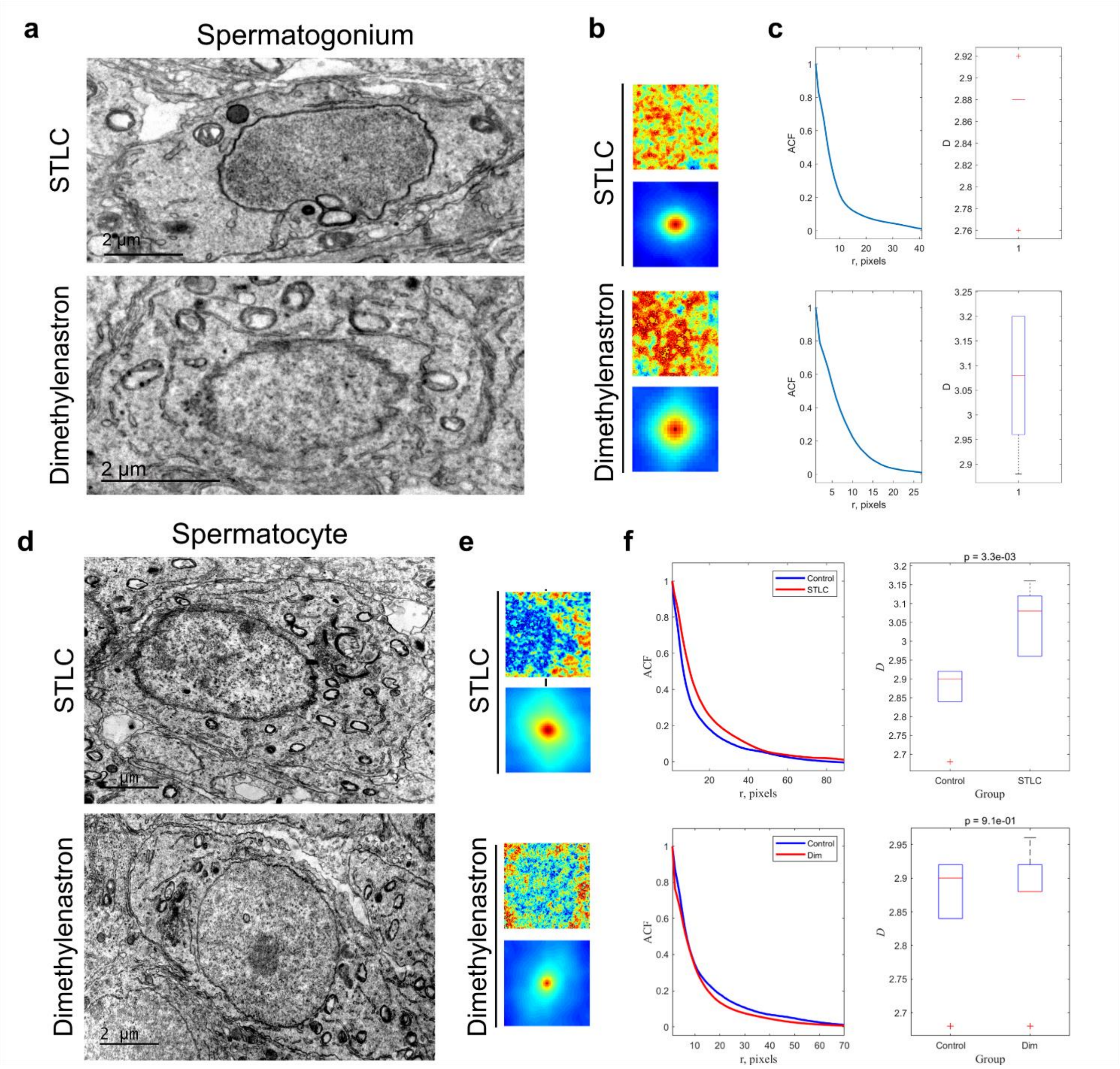

Additional file 1: Fig. S3. The ultrastructure of the spermatogonium and spermatocytes in the STLC and Dimethylenastron group. Related to Fig. 3. **a** Electron microscopic images of the spermatogonium in the STLC and Dimethylenastron groups. Scale bar, 2 μm. **b** The quantifications of chromatin mass density in the spermatogonium (n = 6). **c** Comparisons of the average *ACF* and *D*-value of the spermatogonium in the STLC and Dimethylenastron groups. A boxplot indicated all *D* values corresponding to their correlation functions. **d** Electron microscopic images of the spermatocytes in the STLC and Dimethylenastron group. Scale bar, 2 μm. **e** The quantifications of chromatin mass

density in the spermatocytes in the STLC and Dimethylenastron groups. **f** The diagrams of *D*-values in the STLC and Dimethylenastron groups.

Additional file 1: Fig. S4

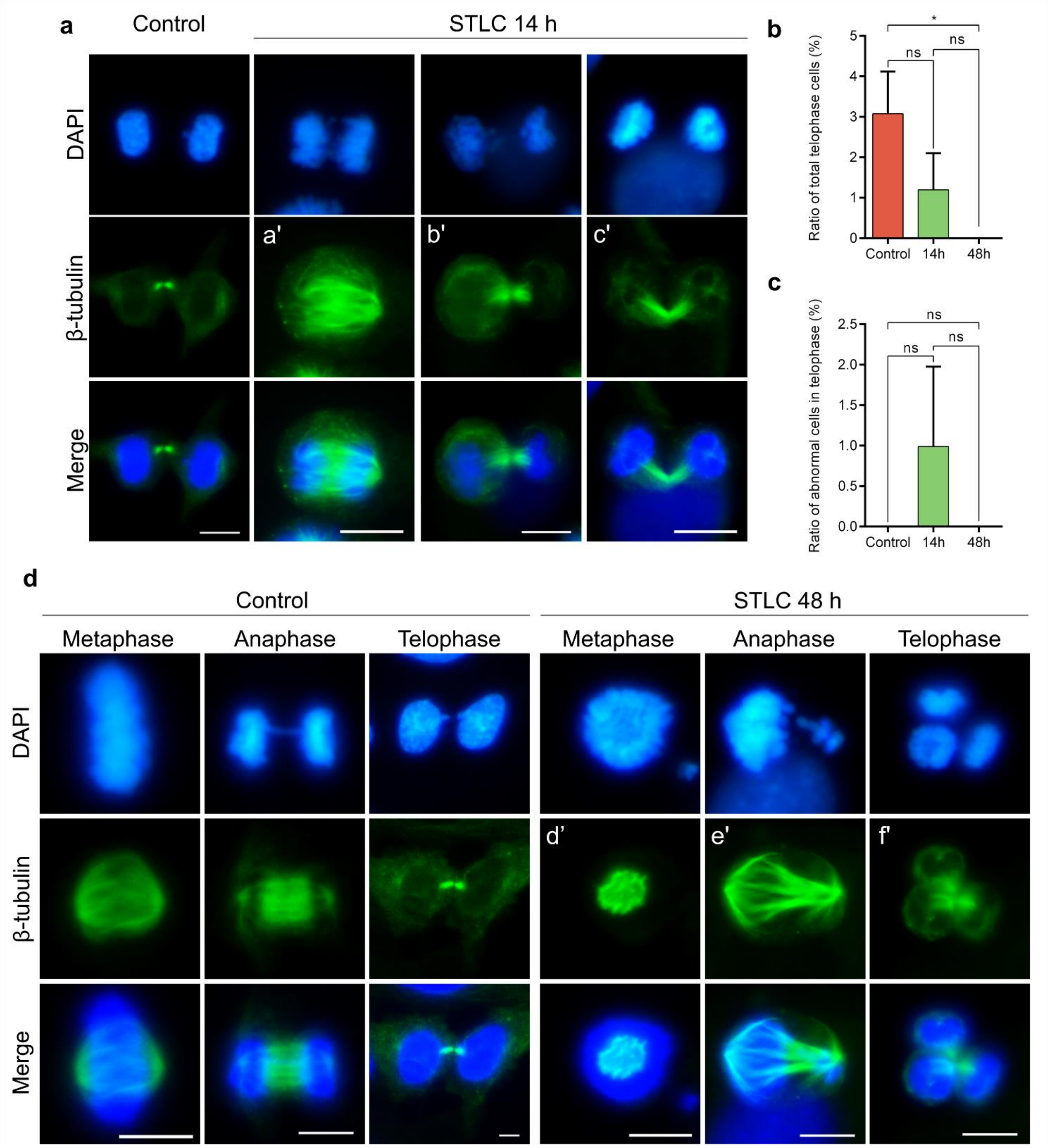

Additional file 1: Fig. S4. Eg5 inhibition results in microtubule disorganization in spindle microtubules in the GC-2 spd cells. Related to Fig. 4. **a** The GC-2 spd cells were cultured with 1 μM STLC for 14 h, leading to chaotic (a'), asymmetrical (b') and folded (c') central spindles during telophase. DAPI (blue), β-tubulin (green). Scale bar, 10 μm. **b** The ratios of total telophase cells in the Control, 14 h STLC and 48 h STLC groups (Control, 3.08 ± 1.04%; 14h, 1.20 ± 0.90%; 48h, 0.00 ± 0.00%). n = 3 per group. **c** The ratios of abnormal cells in telophase in the Control, 14 h STLC and 48

h STLC groups (Control, 0.00 ± 0.00%; 14 h, 0.99 ± 0.99%; 48 h, 0.00 ± 0.00%). n = 3 per group. Student's *t*-test. Error bars, means ± SEM. ns, $p > 0.05$; *, $p < 0.05$. **d** The GC-2 spd cells were cultured with 1 μM STLC for 48 h, leading to monoastral spindle in metaphase (d'), asymmetrical central spindle in anaphase (e') and multipolar central spindle in telophase (f'). DAPI (blue), β-tubulin (green). Scale bar, 10 μm.

Additional file 1: Fig. S5

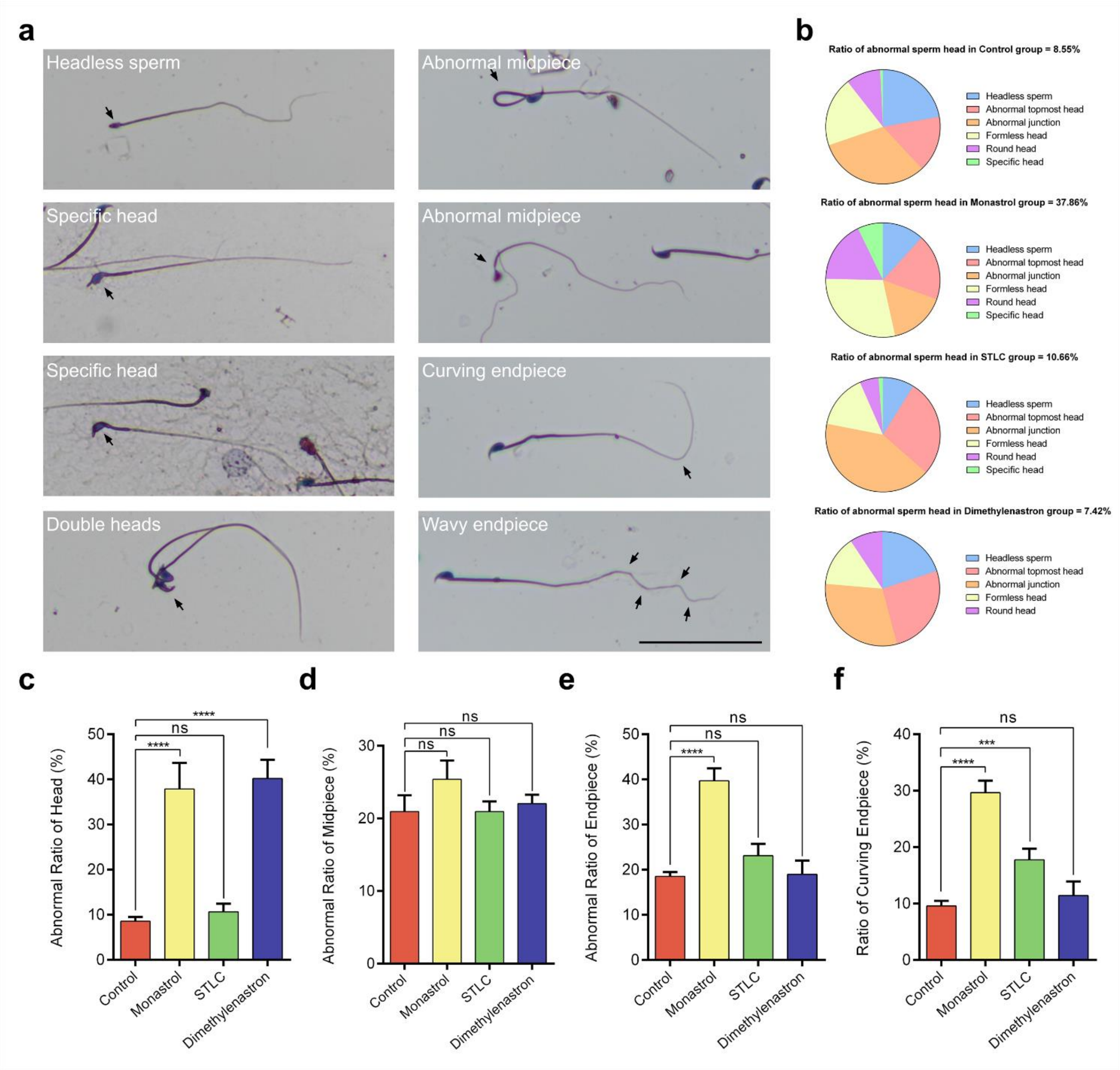

Additional file 1: Fig. S5. Long-term Eg5 inhibition resulted in various types of abnormal sperms. Related to Fig. 7. **a** Detailed morphological characteristics of abnormal sperms. Black arrowheads pointed to the deformities of sperms. Scale bar, 50 μm. **b** The ratios of abnormal sperm head in the Control, Monastrol, STLC and Dimethylenastron groups. (Control, group = 11, n = 101; Monastrol, group = 9, n = 320; STLC, group = 6, n = 80; Dimethylenastron, group = 6, n = 318). **c** The abnormal ratios of head in the Control, Monastrol, STLC and Dimethylenastron groups (Control, 8.55 ± 0.98%; Monastrol, 37.86 ± 5.80%; STLC, 10.66 ± 1.77%; Dimethylenastron, 40.19 ± 4.15%). n=11, 9, 6, 6. **d** The abnormal ratios of midpiece in the Control, Monastrol, STLC and Dimethylenastron groups (Control, 20.93 ± 2.25%; Monastrol, 25.38 ± 2.61%; STLC, 20.94 ± 1.39%; Dimethylenastron, 22.05 ± 1.21%). n = 11, 9, 6, 6. **e** The abnormal ratios of endpiece in Control, Monastrol, STLC and

Dimethylenastron groups (Control, 18.51 ± 0.99%; Monastrol, 39.68 ± 2.75%; STLC, 23.09 ± 2.63%; Dimethylenastron, 18.98 ± 3.05%). n = 11, 9, 6, 6. **f** The ratios of curving endpiece in the Control, Monastrol, STLC and Dimethylenastron groups (Control, 9.57 ± 0.90%; Monastrol, 29.64 ± 2.14%; STLC, 17.75 ± 1.97%; Dimethylenastron, 11.43 ± 2.49%). n = 11, 9, 6, 6. Student's *t*-test. Error bars, means ± SEM. ns, $p > 0.05$; ***, $p < 0.001$; ****, $p < 0.0001$.

Additional file 1: Fig. S6

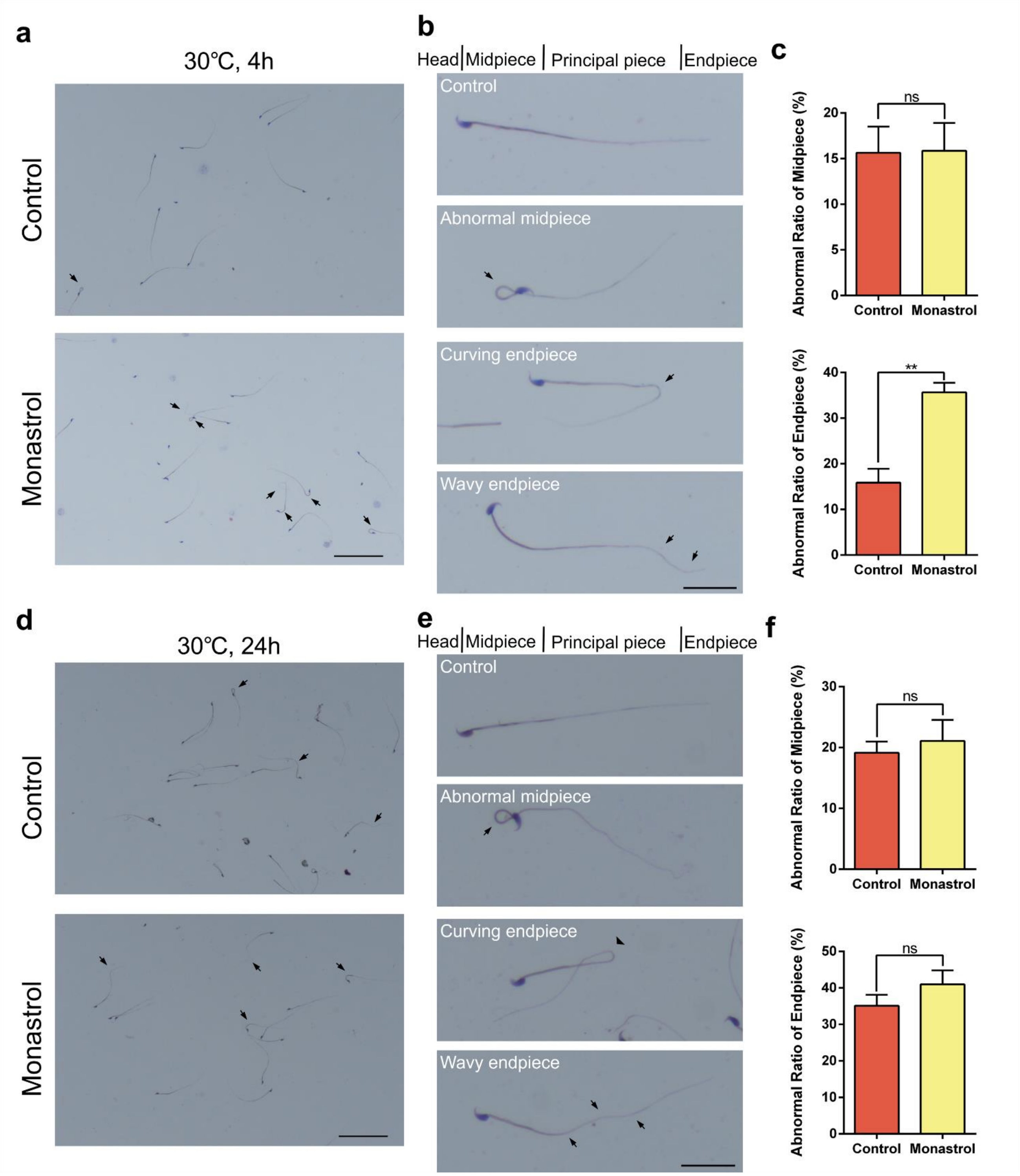

Additional file 1: Fig. S6. Short-term Eg5 inhibition lead to mild phenotypes in mature sperms. Related to Fig. 7. **a, d** HE staining of mature sperms in the Control and Monastrol groups. The semen of untreated 6-month-old mouse was incubated by 50 μM Monastrol at 30℃ for 4 h and 24 h, respectively. Black arrowheads pointed to the deformities of sperms. Scale bar, 100 μm. **b, e** Detailed morphological characteristics of abnormal sperms at 30℃ for 4 h and 24 h. Scale bar, 25 μm. **c** The

abnormal ratios of the midpiece (Control, 15.64 ± 2.87%; Monastrol, 15.87 ± 3.05%) and the endpiece (Control, 15.87 ± 3.05%; Monastrol, 35.65 ± 2.09%) in the Control and Monastrol groups. 30℃ for 4h. n = 3 per group. **f** The abnormal ratios of the midpiece (Control, 19.15 ± 1.83%; Monastrol, 21.09 ± 3.44%) and the endpiece (Control, 35.10 ± 2.99%; Monastrol, 40.97 ± 3.86%) in the Control and Monastrol group. 30℃ for 24 h. n = 3 per group. Student's *t*-test. Error bars, means ± SEM. ns, $p > 0.05$ and **, $p < 0.01$.

Additional file 1: Fig. S7

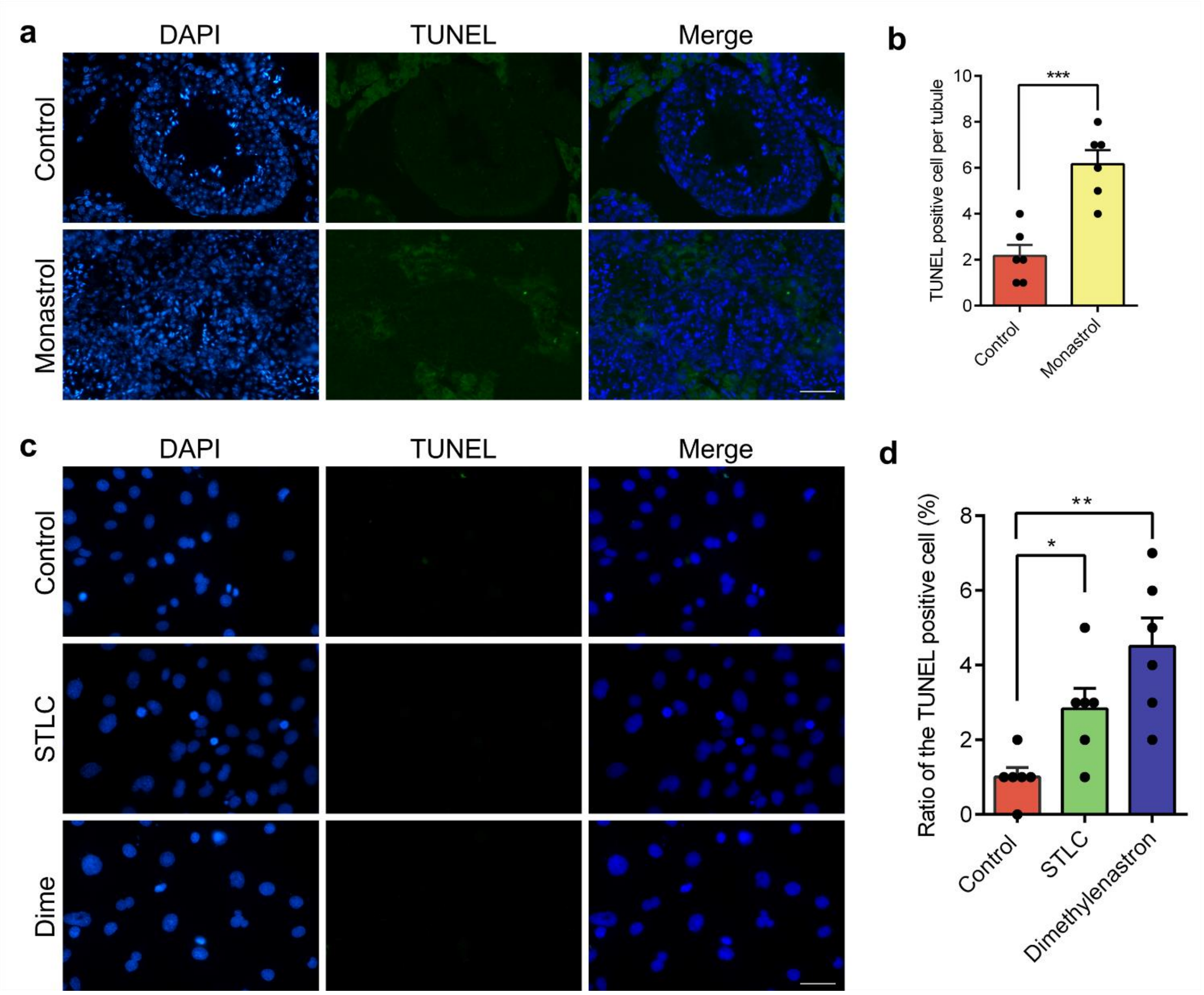

Additional file 1: Fig. S7. Cell apoptosis analyses of seminiferous tubules and GC-2 spd cells after Eg5 inhibition. Related to Fig. 2, 4, 5 and 6. **a** TUNEL analyses of seminiferous tubules treated by Monastrol (50 μM, 2 weeks). **b** Ratio of TUNEL positive cell per tubule. Control, 3.17 ± 0.48; Monastrol, 6.17 ± 0.60. n = 6. Student's *t*-test. Error bars, means ± SEM. ***, $p < 0.001$. **c** TUNEL analyses of GC-2 spd cells cultured by STLC (1 μM, 14 h) and Dimethylenastron (1 μM, 14 h). DAPI (blue), TUNEL (green). Scale bar, 50 μm. **d** Ratio of TUNEL positive cells in the control, STLC and Dimethylenastron groups. Control, 1.00 ± 0.25%; STLC, 2.83 ± 0.54%; Dimethylenastron, 4.50 ± 0.76%. n = 200, group = 6. Student's *t*-test. Error bars, means ± SEM. *, $p < 0.01$; ***, $p < 0.001$.
